# Crystal structure across the β to α phase transition in thermoelectric Cu_2−*x*_Se

**DOI:** 10.1107/S2052252517005553

**Published:** 2017-06-13

**Authors:** Espen Eikeland, Anders B. Blichfeld, Kasper A. Borup, Kunpeng Zhao, Jacob Overgaard, Xun Shi, Lidong Chen, Bo B. Iversen

**Affiliations:** a Center for Materials Crystallography, Department of Chemistry and Interdisciplinary Nanoscience Center (iNANO), Aarhus University, Langelandsgade 140, Aarhus 8000-DK, Denmark; b National Renewable Energy Laboratory, 15013 Denver W Pkwy, Golden, CO 80401, USA; c State Key Laboratory of High Performance Ceramics and Superfine Microstructure, Shanghai Institute of Ceramics, Chinese Academy of Sciences, 1295 Dingxi Road, Shanghai 200050, People’s Republic of China

**Keywords:** thermoelectrics, negative thermal expansion, properties of solids, inorganic materials

## Abstract

The crystal structure uniquely imparts the specific properties of a material, and thus provides the starting point for any quantitative understanding of thermoelectric properties. Cu_2−*x*_Se is an intensely studied high performing, non-toxic and cheap thermoelectric material, and here for the first time, the average structure of β-Cu_2−*x*_Se is reported based on analysis of multi-temperature single-crystal X-ray diffraction data. It consists of Se–Cu layers with additional copper between every alternate layer. The structural changes during the peculiar *zT* enhancing phase transition mainly consist of changes in the inter-layer distance coupled with subtle Cu migration. Just prior to the transition the structure exhibits strong negative thermal expansion due to the reordering of Cu atoms, when approached from low temperatures. The phase transition is fully reversible and group–subgroup symmetry relations are derived that relate the low-temperature β-phase to the high-temperature α-phase. Weak superstructure reflections are observed and a possible Cu ordering is proposed. The structural rearrangement may have a significant impact on the band structure and the Cu rearrangement may also be linked to an entropy increase. Both factors potentially contribute to the extraordinary *zT* enhancement across the phase transition.

## Abstract 2

The average structure of β-Cu_2−*x*_Se is reported based on analysis of multi-temperature single-crystal X-ray diffraction data, and structural changes, including a large negative thermal expansion, across the β to α phase transition are discussed. The structural model also describes well high-resolution synchrotron powder X-ray diffraction data.

## 1. Introduction   

Thermoelectric (TE) materials can directly interconvert heat and electricity, and they potentially provide green sustainable solutions to global energy challenges (Bell, 2008 ▸; Heremans *et al.*, 2013 ▸; Zhang & Zhao, 2015 ▸). However, large scale application requires the development of TE materials made from non-toxic and earth-abundant elements, and this continues to be a key challenge since high performing stable materials typically contain the rare element tellurium (*e.g.* in Bi_2_Te_3_ or PbTe). Cu_2−*x*_Se, *x* ≃ 0, has been intensely studied as a promising cheap TE material (Miyatani, 1973 ▸; Vučić *et al.*, 1982 ▸, 1984 ▸; Liu *et al.*, 2012 ▸, 2013*a* ▸,*b* ▸; Yu *et al.*, 2012 ▸; Brown *et al.*, 2013*a* ▸; Chi *et al.*, 2014 ▸; Mahan, 2015 ▸; Sirusi *et al.*, 2015 ▸; Sun *et al.*, 2015 ▸; Kang *et al.*, 2016 ▸; Brown & Snyder, 2016 ▸), and recently a highly peculiar large increase in thermoelectric performance (*zT*) was observed just prior to the phase transition from the structurally unknown low-temperature β-Cu_2−*x*_Se phase to the cubic high-temperature α-Cu_2−*x*_Se phase (Brown *et al.*, 2013*a* ▸; Liu *et al.*, 2013*b* ▸; Brown & Snyder, 2016 ▸; Kang *et al.*, 2016 ▸). The enhanced performance is too large to be successfully described using conventional band-theory calculations under the normal assumptions (Brown *et al.*, 2013*a* ▸; Mahan, 2015 ▸; Sun *et al.*, 2015 ▸; Kang *et al.*, 2016 ▸), and it has been suggested that the enhancement may be coupled to an entropy increase stemming from a structural rearrangement of the Cu ions (Brown & Snyder, 2016 ▸; Brown *et al.*, 2016 ▸). A detailed understanding of this phenomenon requires knowledge of the crystal structure of the low-temperature β-phase. With regards to nomenclature, there has been some confusion in the literature how to label the low- and high-temperature phase of Cu_2−*x*_Se. In this work we use the original nomenclature introduced by Rahlfs in 1936 (Rahlfs, 1936 ▸).

The maximum efficiency of a thermoelectric generator (Snyder & Toberer, 2008 ▸) is given by the thermoelectric figure of merit *zT*, 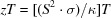, calculated from the Seebeck coefficient (*S*), the electrical conductivity (σ), the thermal conductivity (κ) and the absolute temperature (*T*). An important aspect of the Cu_2−*x*_Se phase transition is that κ is normally determined from the thermal diffusivity, 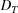, using 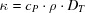, where 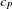 is the specific heat capacity and ρ the density. If a phase transition takes place, 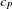 can be interpreted in different ways depending on the order of the transformation (Liu *et al.*, 2013*b* ▸; Brown *et al.*, 2013*a* ▸, 2016 ▸). For Cu_2−*x*_Se the phase transition occurring around 400 K, with the exact value depending on *x*, has been reported both as a first- (Vučić *et al.*, 1984 ▸; Kang *et al.*, 2016 ▸) and a second-order transition (Liu *et al.*, 2013*a* ▸,*b* ▸; Brown *et al.*, 2013*a* ▸; Sirusi *et al.*, 2015 ▸; Mahan, 2015 ▸; Brown & Snyder, 2016 ▸). Without a clear understanding of the nature of the phase transition, the *zT* value of Cu_2−*x*_Se cannot be established correctly.

The development of Cu_2−*x*_Se as a high performing thermoelectric material represents an excellent example of empirical materials optimization in which a fundamental understanding of the system is lacking. In general, the crystal structure imparts the specific intrinsic TE properties of a given material, and thus rational improvement of existing materials, or the design of new materials, necessarily has to be based on knowledge of the atomic structure of the material. Indeed, great progress has been achieved by materials’ genome-like theoretical calculations of transport properties based on known crystal structures. This, for example, led to the discovery of the colossal thermopower in FeSb_2_ (Bentien *et al.*, 2006 ▸, 2007 ▸; Peijie *et al.*, 2009 ▸; Søndergaard *et al.*, 2013 ▸). However, most often new high-performance materials are discovered by highly time-consuming experimental screening, and for known materials the properties are empirically optimized, *e.g.* through systematic substitution or doping. In cases where the detailed crystal structure is unknown the origin of specific properties is unclear and efficient optimization strategies are difficult to develop. In this respect β-Zn_4_Sb_3_ is a good example. This material caused excitement when it was (re)discovered in the 90s (Caillat *et al.*, 1997 ▸), but it was only 10 years later when the detailed crystal structure was solved that the origin of the extraordinary properties could be rationalized (Cargnoni *et al.*, 2004 ▸; Snyder *et al.*, 2004 ▸). Similarly, a very large literature exists on PbTe (Heremans *et al.*, 2008 ▸; Pei *et al.*, 2011 ▸; Biswas *et al.*, 2012 ▸; Christensen *et al.*, 2016 ▸), but only very recently the details of the cation defect and disordered crystal structure were established, potentially paving the way for obtaining a quantitative understanding of its outstanding properties (Božin *et al.*, 2010 ▸; Christensen *et al.*, 2016 ▸). The insufficient structural information regarding the β-Cu_2−*x*_Se phase is not the result of lacking effort. Already in 1936 Rahlfs (1936 ▸) concluded that β-Cu_2−*x*_Se does not adopt the simple antifluorite structure of the α-phase. In 1987 Milat *et al.* proposed a monoclinic super-cell by combining X-ray and electron diffraction (Milat *et al.*, 1987 ▸). Furthermore, the publication contains an overview of 10 different unit cells proposed by previous researchers. A full structure based on single-crystal X-ray diffraction (SCXRD) data has been reported by Gulay *et al.* (2011 ▸), but their proposed structural model did not describe their own diffraction data well resulting in high residuals with *R*_1_ ≃ 0.14. When the structural model of Gulay *et al.* is fitted to the present higher quality SCXRD data with fixed coordinates and atomic displacement parameters (ADPs), (scale factor refinement only), a very high *R*_1_ value of 0.600 is obtained, whereas free refinement of scale, positions and ADPs results in *R*_1_ = 0.294. This shows that the Gulay model is clearly incorrect. In recent years the β-phase has been studied using pair distribution function and density functional theory analysis (Brown *et al.*, 2013*b* ▸; Brown & Snyder, 2016 ▸). Still, as acknowledged widely in the literature, no satisfying structural model has yet been proposed (Milat *et al.*, 1987 ▸; Kashida & Akai, 1988 ▸; Yamamoto & Kashida, 1991 ▸; Frangis *et al.*, 1991 ▸; Gulay *et al.*, 2011 ▸; Nguyen *et al.*, 2013 ▸; Lu *et al.*, 2015 ▸; Brown & Snyder, 2016 ▸). Herein we report the average crystal structure of the low-temperature β-phase of Cu_2−*x*_Se, based on analysis of SCXRD data. The structural changes as the critical temperature is approached are established, and connected to the high-temperature α-phase through symmetry relations. The average structure is further used to model high-resolution synchrotron powder X-ray diffraction (PXRD) data.

**Figure 1:**
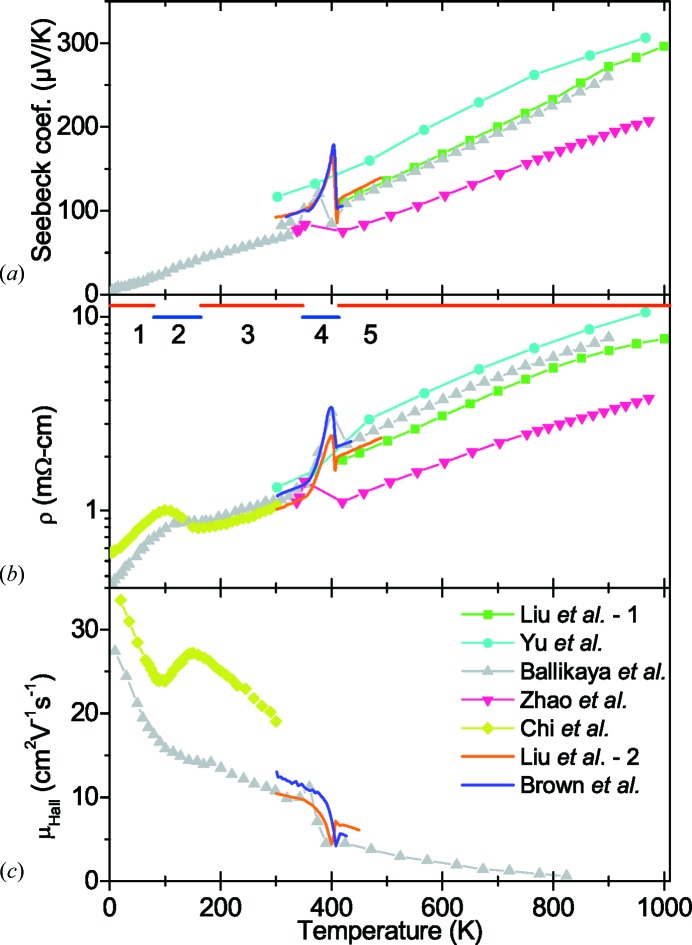
Published thermoelectric data for Cu_2−*x*_Se. (*a*) Seebeck coefficient (*S*), (*b*) resistivity (ρ) and (*c*) hall mobility (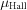) from selected publications to illustrate the trend in the TE properties for Cu_2_Se. The references used are Liu *et al.* (2012 ▸), Yu *et al.* (2012 ▸), Ballikaya *et al.* (2013 ▸), Zhao *et al.* (2015*b* ▸), Chi *et al.* (2014 ▸), Liu *et al.* (2013*b* ▸) and Brown *et al.* (2013*a* ▸).

An overview of the transport properties for Cu_2−*x*_Se, with temperature, is given in Fig. 1 ▸. Overall the properties can be split into five distinct temperature regions (1–5), as indicated. In general, the measured physical properties are in good agreement between studies, taking the difference in copper deficiencies into account (Yu *et al.*, 2012 ▸; Liu *et al.*, 2012 ▸, 2013*a* ▸,*b* ▸; Brown *et al.*, 2013*a* ▸; Ballikaya *et al.*, 2013 ▸; Chi *et al.*, 2014 ▸; Zhao *et al.*, 2015*a* ▸,*b* ▸; Yang *et al.*, 2015 ▸; Kang *et al.*, 2016 ▸). In regions 1, 3 and 5 the TE properties change monotonically in an overall beneficial way, increasing *zT* (Yu *et al.*, 2012 ▸; Liu *et al.*, 2012 ▸; Zhao *et al.*, 2015*a* ▸,*b* ▸). Region 5 corresponds to the known high-temperature α-phase, having attractive TE properties for 0 ≤ *x* < 0.25. The cubic α-phase has been studied as a promising TE material, although since the material is also a superionic conductor this poses an intrinsic challenge with regards to designing TE modules (Brown *et al.*, 2013*b* ▸). In region 2 (80–165 K) abrupt changes in the Hall mobility indicates another phase transition as proposed by Chi *et al.* (2014 ▸), although not observed in the present diffraction data.

In the temperature range 350–410 K, region 4 in Fig. 1 ▸, where the phase transition takes place, most of the physical properties change substantially (Liu *et al.*, 2013*a* ▸,*b* ▸; Brown *et al.*, 2013*a* ▸; Ballikaya *et al.*, 2013 ▸; Chi *et al.*, 2014 ▸; Kang *et al.*, 2016 ▸). The exact temperatures for the onset and termination of the transformation are governed by the copper deficiency with higher copper content narrowing the temperature range from both ends (Takahashi *et al.*, 1976 ▸; Vučić *et al.*, 1984 ▸). Upon heating a change in the slope of σ is observed around 350 K (Liu *et al.*, 2013*a* ▸,*b* ▸; Brown *et al.*, 2013*a* ▸; Ballikaya *et al.*, 2013 ▸; Chi *et al.*, 2014 ▸; Kang *et al.*, 2016 ▸) coinciding with an observed change in the ion conductivity (Miyatani, 1973 ▸; Vučić *et al.*, 1982 ▸; Sirusi *et al.*, 2015 ▸). The increased copper ionic conductivity is linked to structural changes since dilatometry measurements by Vučić *et al.* showed that changes in the thermal expansion also coincide with the change in σ (Vučić *et al.*, 1982 ▸, 1984 ▸; Horvatić & Vučić, 1984 ▸). Very interestingly, negative thermal expansion (NTE) has been observed just prior to the phase transition. Below *T*_c_ there is a sharp drop in κ, 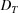, σ, electronic mobility (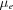), and charge carrier concentration (*n*), whereas there is a peak in *S* (Vučić *et al.*, 1982 ▸; Liu *et al.*, 2013*a* ▸,*b* ▸; Brown *et al.*, 2013*a* ▸; Kang *et al.*, 2016 ▸). In combination this leads to the enhancement of *zT* (Brown *et al.*, 2013*a* ▸; Liu *et al.*, 2013*b* ▸; Brown & Snyder, 2016 ▸; Kang *et al.*, 2016 ▸). Brown *et al.* related the increase in *zT* to the contribution from *S*, and it was believed to stem from an increase in entropy when there is an increase in the mobility of Cu ions (Brown & Snyder, 2016 ▸). Liu *et al.* explains the peak in *zT* by the influence of structural fluctuations on the increase in scattering of charge carriers and phonons (Liu *et al.*, 2013*a* ▸). Mahan has given a theoretical explanation for the peak in *S* as being due to a broadening of the conduction band energy immediately before *T*_c_ (Mahan, 2015 ▸). The broadening can possibly originate from various different effects, but was suggested to ‘probably [be] due to critical fluctuations’ along the same lines as Liu *et al.* (2013*a* ▸,*b* ▸). Yet another explanation was proposed by Sun *et al.*, who showed that a large change in 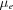 will give rise to an extra contribution to *S* (Sun *et al.*, 2015 ▸). In all these studies a better structural model is clearly needed for β-Cu_2−*x*_Se in order to understand the differences in properties across the phase transition (Brown *et al.*, 2013*a* ▸; Chi *et al.*, 2014 ▸; Sirusi *et al.*, 2015 ▸; Brown & Snyder, 2016 ▸; Kang *et al.*, 2016 ▸). A final point to note is that Cu_2−*x*_Se in the literature often is referred to as a ‘phonon–liquid electron-crystal (PLEC)’ since the disordered nature of the Cu ions in the crystal structure is believed to strongly influence the phonon heat transport (Liu *et al.*, 2012 ▸). If one disregards the region around the phase transition, then the thermal diffusivity is similar in value between the low-temperature and the high-temperature Cu_2−*x*_Se phases (Kang *et al.*, 2016 ▸). This suggests that a complete understanding of the phonon scattering processes in Cu_2−*x*_Se may not have been reached.

## 2. Experimental   

Single crystals of β-Cu_2−*x*_Se were grown using chemical vapor transport (CVT) with iodine and bromine as transport agents. A Cu_2.2_Se precursor was synthesized by direct reaction between elemental copper (99.99%, Alfa Aesar) and selenium (99.999+%, Alfa Aesar) in a sealed quartz ampoule (< 10^−4^ mbar). The ampoule was heated in a vertical tube furnace to 873 K at 100 K h^−1^ and soaked for 10 h for pre-reaction. The furnace was then heated to 1273 K at 100 K h^−1^ and soaked for 42 h for final reaction and homogenization. The resulting ingot was coarsely ground using an agate mortar in air and loaded into two new ampules for CVT crystal growth. The ampoules were cleaned in 20 *M* NaOH at 423 K for 30 min and thoroughly rinsed and soaked overnight in Milli-Q water to improve the quartz surface and reduce the number of nucleation sites. 3.9 g of the ground precursor was loaded in the two ampoules and 20 mg elemental iodine and 32 mg CuBr_2_ was added as transport agents, respectively. The ampoules were sealed with a vacuum (< 10^−4^ mbar) and the precursor material was carefully moved to the end where the ampoule was sealed to allow crystal growth on the treated surface of the ampoule (no visible material was left). The ampoules had an inner diameter of 10 mm and a length of 20 cm and were placed side-by-side in a horizontal two-zone tube furnace (without being in contact) for crystal growth. The growth zone was heated to 1273 K and the source zone to 1223 K and soaked for 3 days. This ensured a clean surface for crystal growth. After the back-transport period the temperature gradient was reversed by first cooling the growth zone to 1223 K and then heating the source zone to 1273 K. After 2 weeks the source zone was cooled to 1225 K and then both the source and growth zones were simultaneously cooled to room temperature. Since only little growth was observed the ampoules were reloaded in the furnace and the process was repeated but with the source and growth zones at 1298 K and 1198 K, respectively. Limited transport was still observed, but significant ripening and crystal growth was obtained in the source zone and a single crystal (0.09 × 0.05 × 0.02 mm^3^) from the hot end of the CuBr_2_ ampoule was selected for SCXRD.

A bulk sample with a total mass of 3.275 g was prepared by first melting a stoichiometric ratio of the elements Cu (shot, 99.999%, Alfa Aesar) and Se (shot, 99.999%, Alfa Aesar) in a pyrolytic boron nitride (P-BN) crucible enclosed in a fused silica tube at 1423 K for 12 h in vacuum, and then slowly cooled down to 923 K in 96 h, where the material was held for 6 days. Finally, the tube was slowly cooled down to room temperature in 50 h.

Multi-temperature SCXRD experiments were carried out on a SuperNova diffractometer from Agilent Technologies, using Mo *K*α radiation (λ = 0.71073 Å), with the temperature regulated by a cryostat from Oxford Cryosystems. Diffracted intensities were collected on a CCD detector and the data integrated and corrected for absorption using *CrysAlisPro* (Xcalibur, 2010 ▸). The structure solution and refinement were carried out with *SHELXT* and *SHELXL*, respectively, using the *Olex*2 GUI (Dolomanov *et al.*, 2009 ▸; Sheldrick, 2015 ▸).

PXRD were collected on the bulk polycrystalline sample at the SPring-8 synchrotron facility (BL44B2 beamline) using a wavelength of λ = 0.500266 (5) Å. Data were collected at 300, 200 and 100 K in that specific order not to induce any thermal hysteresis. The data were analyzed by Rietveld refinements performed in the *MAUD* program (Lutterotti, 2010 ▸). The instrumental contribution to the peak widths was determined from refinement of data collected on a NIST 674b CeO_2_ powder standard, which was also used for wavelength determination. The main phase was modelled using the new average low-temperature structure from SCXRD (see details below), and a secondary cubic (high temperature) phase was included at all temperatures; α-Cu_2−*x*_Se with *x* = 0.25 (ICSD-150758). The cell parameters and the phase fraction were refined for each phase. The β-Cu_2−*x*_Se phase was modeled using an anisotropic particle size, also incorporating microstrain. This advanced model allows the refinement of different peak widths depending on the *hkl* values according to Popa (1998 ▸), which arise from the intrinsic platelet particle shape inherent to most layered structures. The particle shape was extended to *L*_max_ = 8, giving 10 refinable parameters for the particle size. For microstrain, there are 4 parameters available for refinement in the 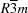 space group. Unit-cell parameters, and the *x* and *y* atomic coordinates were refined and constrained so that Cu2 stayed on the mirror plane. The atomic coordinates were refined for all general Wyckoff sites. The ADPs (*U*_iso_) were refined for all atoms, with Cu1*a* and Cu1*b* constrained to have equivalent ADPs. Furthermore, the summed occupancy for Cu1*a* and Cu1*b* was constrained to be fully occupied. The results from the refinements of β-Cu_2−*x*_Se, including the copper deficient α-Cu_2−*x*_Se phase, are listed in Table S10 of the supporting information. The α-Cu_2−*x*_Se phase was refined using an isotropic particle size model incorporating microstrain. The atomic *x* coordinate for Cu2 on the tetrahedral site was refined, plus all the ADPs and occupancies for Cu1 and Cu2.

## 3. Establishing the average crystal structure of the β-phase   

In order to characterize the crystal structure of the β-phase a suitable single crystal with dimensions 0.09 × 0.05 × 0.02 mm^3^ was selected under a microscope, and SCXRD data were measured on a conventional laboratory (SuperNova) diffractometer. The average structure was solved by intrinsic phasing resulting in the space-group symmetry 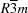 with cell parameters *a* = 4.1227 (8) Å, *c* = 20.449 (6) Å at 100 K, using the hexagonal unit-cell setting. The structure is deposited in the Cambridge Structural Database (1543669–1543677). Many of the proposed unit cells in the literature could be used to successfully index the reciprocal lattice, although only upon integration and comparing equivalent reflections the trigonal symmetry of the high-intensity reflections was established (see the supporting information for more details). The unit-cell parameters are almost identical to those proposed by Vučić *et al.* in 1981 with cell parameters *a* = *b* = 4.10 × 3 Å, *c* = 20.37 × 2 Å, α = γ = 90° and β = 120° found using electron diffraction (Vučić *et al.*, 1981 ▸). Vučić *et al.* as well as previous researchers, also noted the occurrence of supercell reflections (indicated above by the multiplied integers) (Milat *et al.*, 1987 ▸; Frangis *et al.*, 1991 ▸). Superstructure reflections are also observed in the diffraction data obtained in the present work and therefore the structure found by only including the high-intensity reflections can be viewed as an average structure. The low-intensity superstructure reflections contain information about the difference between the superstructure and the proposed average structure. The implications of a superstructure are discussed below and more details concerning refinements of various superstructure models are given in the supporting information. Unfortunately, at this point no accurate description of the full superstructure is available, and the present paper therefore focuses on reporting the average crystal structure.

**Figure 2:**
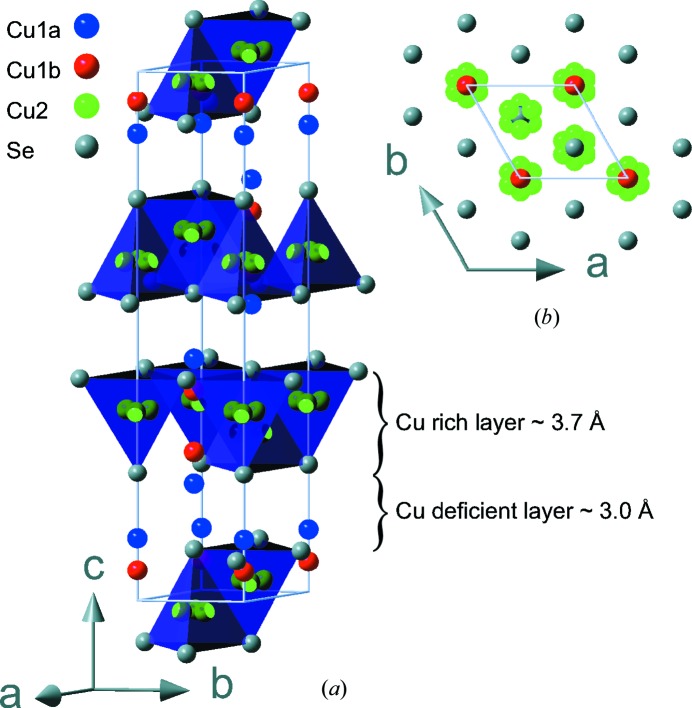
The crystal structure of β-Cu_2−*x*_Se. (*a*) The full unit cell is shown with the copper-rich layers highlighted by purple tetrahedra. (*b*) The structure oriented in the *ab*-plane is shown to the right. Color codes for the atoms are: Cu1*a* (blue), Cu1*b* (red), Cu2 (green) and Se (grey).

The average structure of β-Cu_2−*x*_Se is displayed in Fig. 2 ▸, with selected crystallographic information listed in Table 1 ▸. The structure consists of hexagonal close packed (c.p.) Se layers stacked along the *c*-axis, with Cu atoms located slightly above and below the c.p. layers, with additional disordered copper located between every alternate layer. The asymmetric unit consists of a single Se position and two disordered copper sites (Cu1 and Cu2). Se and Cu1 are both located on threefold rotation axes with the Cu1 site splitting into two (Cu1*a* and Cu1*b*) positions located slightly above and below the Se layer. Cu2 is slightly shifted away from the threefold rotation axis. Freely refining occupancies of the two copper sites (Cu1 and Cu2) resulted in an overall stoichiometry of Cu_1.94 (2)_Se at 100 K. However, the stoichiometry derived from SCXRD analysis is generally not well determined, in particular for disordered structures, since the occupancies correlate with ADPs. The elemental composition was therefore measured using energy dispersive X-ray spectrometry (EDX) on a single crystal from the same batch revealing a composition of Cu_1.95 (13)_Se. In all the following structural refinements the Cu content was fixed to the value derived from EDX. The crystal structure has two different inter-layer distances, 3.067 (1) Å and 3.742 (1) Å at 295 K, with the longer distance being between layers containing the disordered copper, Fig. 2 ▸. The present structural motif is in good agreement with three previous structural models suggested from theoretical calculations, although they propose different unit-cell parameters (Fig. S14) (Liu *et al.*, 2013*b* ▸; Lu *et al.*, 2015 ▸). As theoretical calculations in general do not accommodate disordered atomic positions, the three theoretical models differ in their arrangement of Cu atoms. Despite this the calculated energies for the three phases are almost identical, indicating that different ordering of the Cu atoms has similar energies. This corroborates that the Cu atoms are disordered on the length scale probed in our diffraction experiment. However, as discussed below a superstructure with ordered Cu atoms might possibly be found using a large supercell. The diffraction experiments carried out on the high-temperature α-phase showed the well known cubic structure with *a* = 5.8538 (3) Å at 400 K. The structure consists of cubic close-packed Se with disordered Cu located at the tetrahedral sites, splitting in two positions, the tetragonal site and one slightly shifted along the body-diagonal. In the structural model the ADPs of the two Cu sites are constrained to have the same value and the overall stoichiometry fixed to Cu_1.95_Se.

## 4. Structural changes across the phase transition in Cu_2−*x*_Se   

In order to characterize the structural changes leading up to the phase transition diffraction data were collected at multiple temperatures. Data were first collected at 100 K, 295 K and 400 K. At 400 K the phase transition is complete and data were then measured at 355 K, 380 K, 365 K and 372 K in that sequence determining *T*_c_ to lie somewhere between 372 K and 380 K for this specific sample. The phase transition was found to be completely reversible without noticeable degradation in the crystal quality. In the high-temperature α-phase Cu_2−*x*_Se has the antifluorite structure with cubic close packed Se atoms and with disordered Cu atoms at all the tetrahedral sites, and along the body diagonal. It should be noted that it has been debated whether the Cu disorder is static or dynamic (Danilkin *et al.*, 2012 ▸).

**Figure 3:**
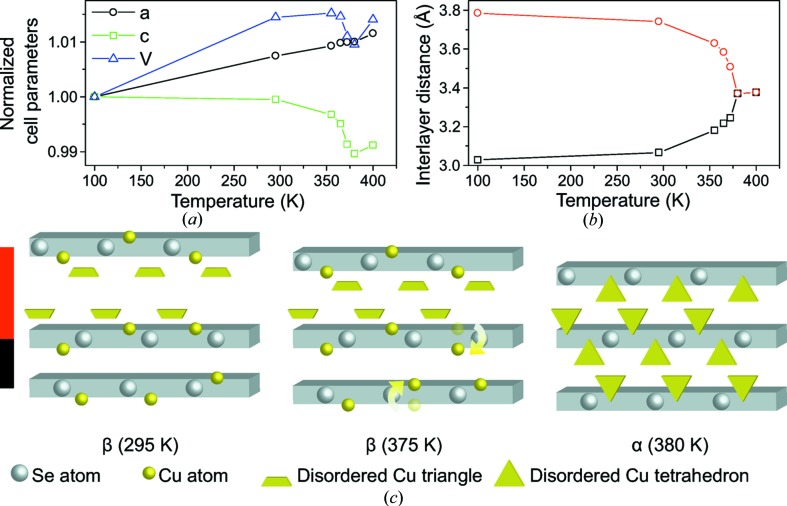
Multi-temperature structural data for Cu_2−*x*_Se. (*a*) Normalized cell parameters for Cu_1.95_Se. At 100 K the hexagonal cell parameters are *a* = 4.092 (1) Å, *c* = 20.459 (9) Å and *V* = 296.7 (2) Å^3^. (*b*) Changes in inter-layer distances as a function of temperature, red is the long distance and black short. The two last data points in both figures are from the cubic α-phase. All uncertainties are smaller than the size of the symbols. (*c*) Three stages of the copper movement from the thick copper rich layer (red) to the thinner copper deficient layer (black). Copper atoms are represented as spheres, with trapeze symbols indicating Cu sites disordered about a threefold rotation axis, while triangles indicate disordered copper tetrahedra in the α-phase.

Normalized unit-cell parameters as a function of temperature are shown in Fig. 3 ▸(*a*) with the last two data points being from the high-temperature α-phase. The data clearly show that there is NTE just before the phase transition. This has also been observed using dilatometry (Vučić *et al.*, 1984 ▸), and from 355 K to 372 K the unit-cell volume decreases 0.41 (3) %. After the phase transition the unit-cell volume starts to increase with temperature. The NTE is highly anisotropic being caused uniquely by a decrease in length of the *c*-axis, which is directly related to changes in the two inter-layer distances plotted in Fig. 3 ▸(*b*). The linear expansion along the *c*-axis, α_c_, from 355 K to 372 K amount to −3.2 × 10^−4^ K^−1^. In this short temperature range the negative expansion is comparable to the expansion found in colossal NTE materials, *e.g.* BiNiO_3_ (Azuma *et al.*, 2011 ▸). The *a*- and *b*-axes increase linearly in the measured temperature range. The distance between the layers containing the interstitial copper atoms (Cu2) drastically shortens near *T*_c_. The shrinkage is almost compensated by an increased distance between the empty c.p. layers without interstitial copper. It is evident that up to the phase transition the two distinct inter-layer distances continuously approach the same value as observed in the cubic α-phase, Fig. 3 ▸(*b*). The changes in inter-layer distances are correlated with a significant Cu migration from the copper rich to the copper deficient layer, as illustrated in Fig. 3 ▸(*c*). From 295 K to 372 K an amount of electron density corresponding to ¼ Cu atom migrates from Cu1*b* to the Cu1*a* position, while the Cu occupancies at the disordered Cu2 site remain constant up to *T*_c_ (see Fig. S12). It should be noted that the Cu occupancies correlate with its ADPs, and care should to be taken when interpreting absolute values of occupancies. However, since the observed *changes* in occupancies are large compared with the uncertainties, and the total occupancy at the Cu1 site remain constant (when freely refined) up to *T*_c_, the migration is credible.

## 5. Symmetry relations between the α- and β-phase   

Recently Brown *et al.* (2016 ▸) suggested that the phase transition in Cu_2−*x*_Se for *x* ≃ 0 is of second order or continuous, while other studies have reported it as a first-order transition (Brown *et al.*, 2013*a* ▸; Vučić *et al.*, 1984 ▸; Kang *et al.*, 2016 ▸). According to Landau Theory, during a continuous phase transition the space group of one phase must be a subgroup of the space group of the other phase (Müller, 2013 ▸). If a group–subgroup symmetry relation exists, *i.e.* if it is possible to relate the symmetry of the high-temperature α-phase to the symmetry of the low-temperature β-phase, then we cannot exclude that the phase transition is of second order. Furthermore, and more importantly for this work, the derivation of such symmetry relations adds credibility to the structural model.

**Figure 4:**
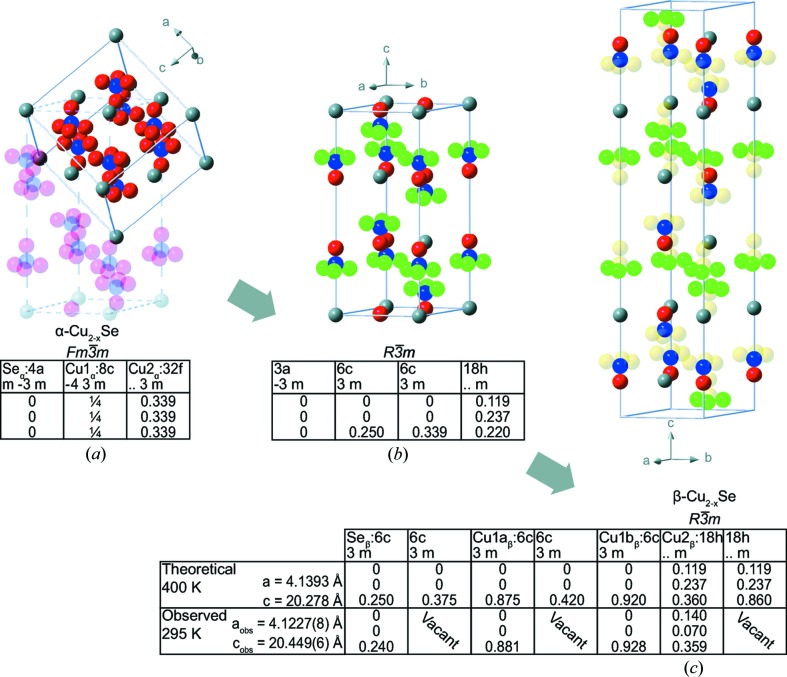
Symmetry-related phase transitions in Cu_2−*x*_Se. (*a*) The cubic α-structure. Se_α_ (grey, 4*a*, 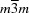), Cu1_α_ (blue, 8*c*, 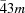), Cu2_α_ (red, 32*f*, *3m*). (*b*) Intermediate structure with symmetry reduction leading to split Cu positions. Se (grey, 3*a*, 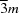), Cu1_α_ (blue, 6*c*, 3*m*), Cu2*a*_α_ (red, 6*c*, 3*m*), Cu2*b*_α_ (green, 18*h*, m). (*c*) The β-Cu_2−*x*_Se structure after symmetry lowering from the intermediate phase. Se_β_ (grey, 6*c*, 3*m*), Cu1*a*_β_ (blue, 6*c*, 3*m*), Cu1*b*_β_ (red, 6*c*, 3*m*), Cu2_β_ (green, 18*h*, m). The transparent yellow atoms are the resulting vacant sites.

In the following, such symmetry relations are derived leading to exceptional good agreement between predicted and observed atomic positions. The α- and β-Cu_2−*x*_Se structures are found to be related through two simple symmetry reduction steps as illustrated in Fig. 4 ▸ and the full Bärninghausen-tree is given in Fig. S13. The first step involves the loss of cubic symmetry. The α-Cu_2−*x*_Se structure has the cubic space-group symmetry 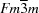, with *a* = 5.8538 (3) Å at 400 K (see Table 1 ▸). The loss of cubic symmetry results in an intermediate trigonal structure with space group, 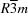, as depicted in Fig. 4 ▸(*b*). In this structure the disordered Cu position is split into two distinct sites, with only ¼ of the positions retaining the threefold rotation symmetry. No indication of this proposed intermediate structure is found in the present diffraction data.

The second symmetry reduction leading to the low-temperature β-phase is a doubling of the *c*-axis. Here each of the three distinct copper positions from the intermediate structure are split into two crystallographically independent positions, while retaining their site symmetries. The atomic positions calculated by applying the symmetry reductions to positions of the cubic structure (at 400 K) can now be compared directly with the observed experimental positions for the β-structure, Fig. 4 ▸(*c*). Half of the Cu positions are vacant and this results in the observed layered structure with alternating Cu rich and Cu deficient layers. Furthermore, the Se position, which now has an unrestricted *z* coordinate, is shifted by 0.2 Å. This results in the two different inter-layer distances of 3.1 and 3.7 Å (at 295 K). Interestingly, the Cu1 position is shifted only 0.2 Å, while the disordered Cu2 site is shifted 0.7 Å when going through the phase transition closing in on the threefold rotation axis. This nicely illustrates that our single crystal of Cu_2−*x*_Se undergoes an order–disorder transition in combination with a displacive phase transition. The widely assumed order–disorder nature of the transformation confirmed here should be seen in relation to the α-structure, *i.e.* the β-structure is more ordered than the α-structure, despite both structures containing disordered Cu positions.

## 6. Superstructure considerations   

Although a full structure describing all superstructure peaks has not yet been found, it is informative to illustrate the discrepancies in the proposed models. A large monoclinic supercell with cell parameters, *a* = 7.154 (2) Å, *b* = 12.415 (3) Å, *c* = 27.440 (7) Å and β = 94.69 (2)° can index most of the observed superstructure reflections at 295 K. This cell indexes the same reflections as the unit cell proposed by Gulay *et al.* (2011 ▸). Integration using the large supercell also gives a reasonable *R*_int_ value of 0.085, when using the *C*2/*c* space-group symmetry. Owing to the similar unit cell proposed by Gulay *et al.* their proposed structural model was refined against our diffraction data. However, it quickly became clear that the Gulay structural model does neither fit the superstructure nor the high-intensity reflections resulting in very high residual values with *R*_1_ = 0.570 and 0.610, respectively (scale factor refinement only). The *R*_1_ values were calculated by dividing reflections into two groups; the high-intensity main reflections which are reflections also indexed by the average trigonal structure, and the superstructure reflections which are all the reflections indexed by the monoclinic supercell but not indexed by the average trigonal structure (see the supporting information §S3 and §S4 for more information). The integration using the monoclinic unit cell results in 236 main reflections and 2226 superstructure reflections at 295 K. Out of 2226 superstructure reflections 878 have *I*/σ > 2. The reason why the 295 K data are used instead of the 100 K data is that the diffraction data obtained at 100 K are slightly twinned.

**Figure 5:**
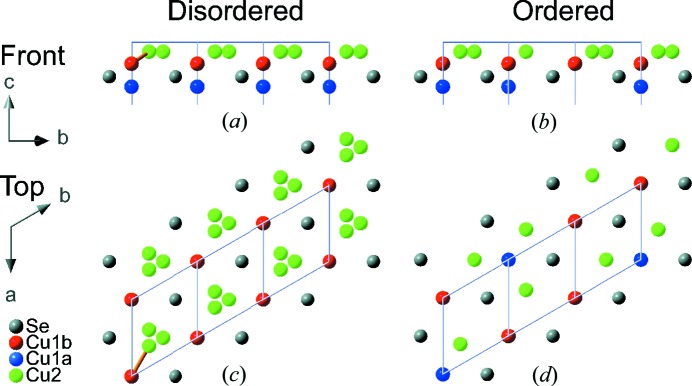
Proposed ordering of Cu atoms in a superstructure. The figure shows one c.p. layer where the copper atoms are disordered in the average structure [(*a*) and (*c*)] and the proposed superstructure [(*b*) and (*d*)]. (*a*) and (*b*) shows the *ab*-plane for disordered and ordered copper sites, respectively. In (*c*) and (*d*) the structure is seen from the top along the *c*-axis for disordered and ordered copper sites, respectively. Se (grey), Cu1*a* (blue), Cu1*b* (red) and Cu2 (green). The orange bond illustrates the unphysical short Cu1*b*—Cu2 contact.

In reciprocal space the *b* ^*^-axes of the monoclinic supercell and the average cell are overlapping and oriented in the same direction, but the *b*-axis of the monoclinic supercell is three times as long as for the average cell. This relation might qualitatively be explained by taking a closer look at the average structure, as a random distribution of Cu atoms on the two disordered Cu sites in the average structure leads to unrealistically short Cu—Cu distances. The short Cu—Cu distances are shown in Fig. 5 ▸ together with a possible local ordering of the Cu atoms in the *ab*-plane. Looking down the *c*-axis including only a single c.p. layer and the closest Cu sites, the shortest Cu1*b*—Cu2 distance of 2 Å cannot exist in the structure (shown as an orange bond in Fig. 5 ▸). If we remove the Cu1*b* site and keep the Cu1*a* atom located right on the other side of the c.p. layer, a realistic Cu1*a*—Cu2 distance of 2.7 Å is obtained. At 295 K the occupancies of Cu1*b* and Cu1*a* have a ratio of 2:1. This agrees with the copper ordering shown in Figs. 5 ▸(*b*) and (*d*), containing no short Cu—Cu distances. If the ordering is of long-range, this would result in a tripling of the *a*/*b*-axis. The described ordering of Cu sites has been incorporated into the monoclinic superstructure and refined against the diffraction data. However, the model did not fit the diffraction intensities with an overall *R*_1_ of 0.378 for all reflections. From the constructed electron density difference map (Fig. S10) it is evident that in order to improve the model disordered Cu sites still need to be introduced into the structural model. By splitting 10 out of 12 Cu sites and incorporating anisotropic ADPs the fit improved considerably resulting in an overall *R*_1_ of 0.119. This superstructure model accurately describes the main peak intensities (*R*_1_ = 0.062), but the superstructure reflections have quite a poor residual (*R*_1_ = 0.253). In order to progress and obtain a better structural model than the average structure reported here, better *I*/σ ratios for all superstructure reflections are needed. We have repeatedly tried to measure data using high brilliance synchrotron facilities, but in all cases poor crystal quality has led to worse data than reported here.

Upon cooling the α-phase, weak supercell reflections emerge already at 372 K. When the sample is cooled to 100 K the intensity of the supercell reflections increases relative to the main reflections with a factor of ~5 (Table S9). This indicates that the superstructure is present up to the phase transition, but that thermal motion blurs the difference between the superstructure and the reduced cell of the average structure. The intensities of the supercell reflections at different temperatures are depicted in Fig. S8. Short-range order in the structure is also evident directly in the collected diffraction data as diffuse scattering. The diffuse scattering is most prominent along the reciprocal c*-axis.

## 7. Rietveld refinements of synchrotron PXRD data   

**Figure 6:**
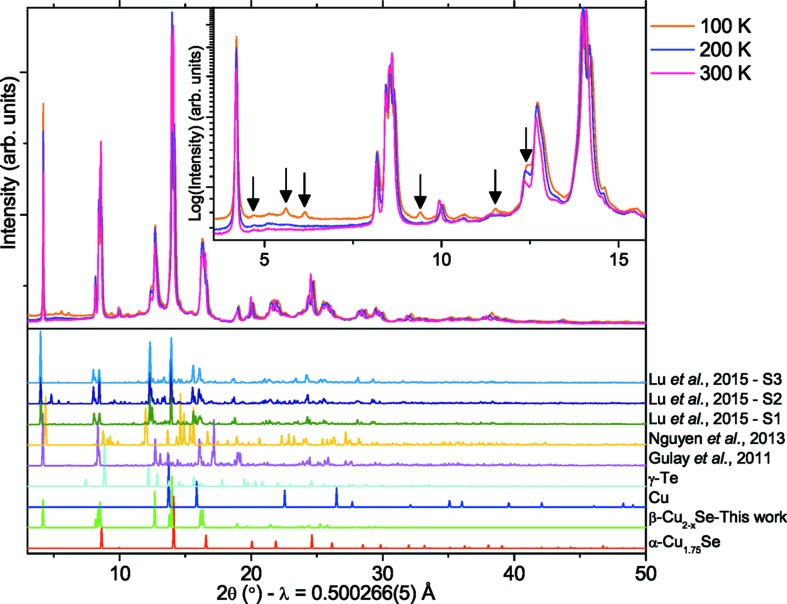
Synchrotron PXRD for the polycrystalline Cu_2−*x*_Se sample. The insert is a zoom of the region where the supercell peaks are observed most clearly, marked by arrows. The theoretical diffraction patterns for the previously reported and related structures are shown in the lower panel for comparison. The α-Cu_1.75_Se phase is ICSD-#150758.

To study the general applicability of the average structure of β-Cu_2−*x*_Se, synchrotron PXRD data were measured on a polycrystalline bulk sample at three different temperatures, 100 K, 200 K and 300 K. The sample was found to also contain about 20 vol%, 25 vol% and 40 vol% of the cubic α-phase at 100, 200 and 300 K, respectively. The collected data are shown in Fig. 6 ▸ together with theoretical PXRD patterns for the structures published in the literature (Lu *et al.*, 2015 ▸; Nguyen *et al.*, 2013 ▸; Gulay *et al.*, 2011 ▸). Superstructure peaks are indicated by black arrows. All the diffraction patterns show similar features with certain groups of reflections in the vicinity of the reflections from the experimental data, but none of the previously proposed structural models result in satisfactory Rietveld refinements (*R*_B_ < 0.02).

In Fig. S14 the different structures are compared and even though the unit-cell parameters differ the structural similarities are obvious. The disorder on the copper sites together with higher symmetry is what sets the present average structure apart from the previously reported structures. By using the proposed average structure of β-Cu_2−x_Se to model the PXRD data, refinements with low residuals were achieved. The resulting fits are plotted in Fig. S15 with the fitting parameters tabulated in Table S10. The average model fits the data extremely well, despite not including the weak superstructure features. The peak broadening is quite severe and needs to be well described in order to obtain good data fits. This is incorporated by using an anisotropic particle size model by Popa (1998 ▸). During the cooling process, some of the α-phase transforms into the β-phase, and at the same time the copper content in the remaining α-phase decreases, giving a composition of Cu_1.81_Se, Cu_1.71_Se and Cu_1.68_Se for the cubic phase at 300, 200 and 100 K, respectively. The change in phase fraction and the copper content in the cubic phase indicates that copper is still quite mobile, even at low temperatures.

Upon cooling, the Cu in the β-phase moves from the copper-deficient layer Cu1*a* position to the C1*b* position in the copper-rich layer, as was also observed in SCXRD refinements (Table S10). The remaining parameters behave as expected. With the aid of the average structure new investigations of the phase diagram of Cu and Se are encouraged, since accurate phase fractions can now be extracted using Rietveld refinements.

## 8. Concluding remarks   

Many structures related to Cu_2−*x*_Se show similar physical properties such as *A*_2_ *X*, with *A* = Cu, Ag and *X* = S, Se, Te, but the *zT* enhancement near a phase transition is quite a new phenomenon. A similar but smaller *zT* enhancement was observed in the ion-conducting material AgCrSe_2_ near *T*_c_ (Gascoin & Maignan, 2011 ▸). Interestingly, AgCrSe_2_ has a similar structure to β-Cu_2−*x*_Se, although in the former the phase transition does not result in a lattice distortion (Damay *et al.*, 2016 ▸). In both the LT (*R*3*m*) and HT (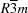) structure AgCrSe_2_ forms a layered structure with Cr and Ag positioned in between adjacent c.p. Se layers at the octahedral and tetrahedral sites, respectively (Van Der Lee & Wiegers, 1989 ▸). In AgCrSe_2_, the Seebeck enhancement is presumably related to a redistribution of the Ag ions during the phase transition. If the Seebeck enhancement in Cu_2−*x*_Se up to the phase transition is related to the entropy carried by the Cu ions, then substituting Ag into the structure should increase the entropy carried by the ions and increase the effect. This is indeed observed for Cu_1.97_Ag_0.03_Se (Brown *et al.*, 2013*a* ▸).

Brown *et al.* (2016 ▸) showed that the peak in *zT* is due to the peak in *S* since 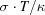 decreases and shows a minimum at the phase transition. The peak in *S* must therefore more than compensate for this minimum to result in the observed peak in *zT*. This has been interpreted by Brown *et al.* (Brown *et al.*, 2013*a* ▸; Brown & Snyder, 2016 ▸) as a coupling of the changing entropy of the Cu ions to the charge carriers across the phase transition, thus invoking the interpretation of the Seebeck coefficient as the entropy transported per charge carrier. The band structure was assumed to vary smoothly across the phase transition, hence disfavoring an explanation based on specific changes to the band structure.

In our study we observe a significant NTE and continuous changes in the inter-layer distances leading up to *T*_c_. A decreasing unit-cell size can lead to an increased band gap, and a change in the inertial effective mass of the charge carriers (Zeier *et al.*, 2016 ▸). In the single parabolic band model an increase in the effective mass will lead to a decrease in the electrical mobility and a peak in the resistivity (assuming constant carrier concentration) as observed in Fig. 1 ▸. In this picture the increase in the Seebeck coefficient may be explained by the increased effective mass. Mahan found that the increase in *S* can be explained by a broadening of the conduction band energy (Mahan, 2015 ▸). He suggested several possible explanations for such broadening including cation disordering, inhomogenous broadening or critical fluctuations. The present average structure may facilitate further work leading to an unambiguous explanation for the extraordinary properties of Cu_2−*x*_Se.

In summary, we report the average structure of the low-temperature β-phase of Cu_2−*x*_Se based on SCXRD, and the structure is shown to also provide an excellent fit to synchrotron PXRD using the Rietveld method. A potential ordering of the Cu atoms has been proposed based on the observation of weak superstructure reflections. As the temperature approaches the transition temperature the superstructure reflections disappear, indicating that at temperatures close to the transition temperature the average structure reported is a good approximation. The structural changes presented are likely important for the peculiar enhancement of *zT* during the β-to α-phase transition, which is governed by a redistribution of the Cu atoms. The widely assumed order–disorder character of the transition is confirmed, and specific group–subgroup symmetry relations were derived for the structures across the completely reversible phase transition.

## Supplementary Material

Crystal structure: contains datablock(s) global, Cu1p95Se_CVT1_100K, Cu1p95Se_CVT1_295K, Cu1p95Se_CVT1_355K, Cu1p95Se_CVT1_365K, Cu1p95Se_CVT1_372K, Cu1p95Se_CVT1_380K, Cu1p95Se_CVT1_400K, Cu2-xSe_CVT2_100K_afterheatingto450K, Cu2-xSe_bulk_100K. DOI: 10.1107/S2052252517005553/lc5071sup1.cif

Structure factors: contains datablock(s) Cu1p95Se_CVT1_100K. DOI: 10.1107/S2052252517005553/lc5071Cu1p95Se_CVT1_100Ksup2.fcf

Structure factors: contains datablock(s) Cu1p95Se_CVT1_295K. DOI: 10.1107/S2052252517005553/lc5071Cu1p95Se_CVT1_295Ksup3.fcf

Structure factors: contains datablock(s) Cu1p95Se_CVT1_355K. DOI: 10.1107/S2052252517005553/lc5071Cu1p95Se_CVT1_355Ksup4.fcf

Structure factors: contains datablock(s) Cu1p95Se_CVT1_365K. DOI: 10.1107/S2052252517005553/lc5071Cu1p95Se_CVT1_365Ksup5.fcf

Structure factors: contains datablock(s) Cu1p95Se_CVT1_372K. DOI: 10.1107/S2052252517005553/lc5071Cu1p95Se_CVT1_372Ksup6.fcf

Structure factors: contains datablock(s) Cu1p95Se_CVT1_380K. DOI: 10.1107/S2052252517005553/lc5071Cu1p95Se_CVT1_380Ksup7.fcf

Structure factors: contains datablock(s) Cu1p95Se_CVT1_400K. DOI: 10.1107/S2052252517005553/lc5071Cu1p95Se_CVT1_400Ksup8.fcf

Structure factors: contains datablock(s) Cu2-xSe_CVT2_100K_afterheatingto450K. DOI: 10.1107/S2052252517005553/lc5071Cu2-xSe_CVT2_100K_afterheatingto450Ksup9.fcf

Structure factors: contains datablock(s) Cu2-xSe_bulk_100K. DOI: 10.1107/S2052252517005553/lc5071Cu2-x5e_bulk_100Ksup10.fcf

Supporting figures and tables. DOI: 10.1107/S2052252517005553/lc5071sup12.pdf

CCDC references: 1543669, 1543670, 1543671, 1543672, 1543673, 1543674, 1543675, 1543676, 1543677

## Figures and Tables

**Table:** The structure solutions and refinements were carried out with *SHELXT* and *SHELXL*, respectively. The empirical multi-scan correction was used to account for absorption.

		β-Cu_2−*x*_Se		α-Cu_2−*x*_Se	
Chemical formula†		Cu_1.95_Se		Cu_1.95_Se	
Space group		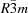 ; H		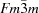	
Temperature (K)		295		400	
*a* (Å)		4.1227 (8)		5.8538 (3)	
*c* (Å)		20.449 (6)		–	
*V* (Å^3^)		301.0 (2)		200.59 (3)	
*Z*		6		4	
ρ_calc_ (g cm^−3^)		6.711		6.707	
μ (mm^−1^)		38.277		38.257	
*F*(000)		543		362	
(sin θ/λ)_max_ (Å^−1^)		0.62		0.62	
*T*_min_, *T*_max_		0.17, 1.00		0.32, 1.00	
*N*_Tot,obs_		809		277	
*N*_Uniq,obs_		102		22	
*N*_Parameters_		19		6	
GOF		1.210		1.261	
*R*_int_		0.075		0.079	
*R*_1_, *R*_1_[*F* ^2^ > 2σ(*F* ^2^)]		0.061, 0.059		0.043, 0.043	
*wR*_2_, *wR*_2_[*F* ^2^ > 2σ(*F* ^2^)]‡		0.158, 0.156		0.087, 0.087	
Δρ_max_, Δρ_min_ (e Å^−3^)		1.33, −1.39		0.50, −0.91	

**Table:** 

	Position (*x*, *y*, *z*)	Wyckoff position	Occupancy	*U*_eq_ (Å^2^)
β-Cu_2−*x*_Se (295 K)				
Se	[0, 0, 0.2416 (1)]	6*c*	1	0.025 (1)
Cu1*a*	[0, 0, 0.119 (1)]	6*c*	0.34 (3)	0.045 (5)
Cu1*b*	[0, 0, 0.0720 (8)]	6*c*	0.64 (3)	0.054 (5)
Cu2	[0.263 (1), 0.526 (2), 0.0255 (3)]	18*h*	0.322 (6)	0.051 (3)
				
α-Cu_2−*x*_Se (400 K)				
Se	(0, 0, 0)	4*a*	1	0.042 (2)
Cu1§	(1/4, 1/4, 1/4)	8*c*	0.61 (2)	0.071 (4)
Cu2	[0.339 (4), 0.339 (4), 0.339 (4)]	32*f*	0.091 (6)	0.071 (4)

† The copper content is restrained to the result from the elemental analysis.

‡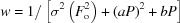, 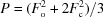. *a* = 0.098 and *b* = 11.93 at 295 K. *a* = 0 and *b* = 16.35 at 400 K.

§ In the α-Cu_2−*x*_Se refinement the copper atoms are constrained to having the same ADPs.

## References

[bb1] Azuma, M., Chen, W., Seki, H., Czapski, M., Olga, S., Oka, K., Mizumaki, M., Watanuki, T., Ishimatsu, N., Kawamura, N., Ishiwata, S., Tucker, M. G., Shimakawa, Y. & Attfield, J. P. (2011). *Nat. Commun.* **2**, 347. 10.1038/ncomms1361 PMC3156814 21673668

[bb2] Ballikaya, S., Chi, H., Salvador, J. R. & Uher, C. (2013). *J. Mater. Chem. A*, **1**, 12478–12484.

[bb3] Bell, L. E. (2008). *Science*, **321**, 1457–1461. 10.1126/science.1158899 18787160

[bb4] Bentien, A., Johnsen, S., Madsen, G. K. H., Iversen, B. B. & Steglich, F. (2007). *Europhys. Lett.* **80**, 17008.

[bb5] Bentien, A., Madsen, G. K. H., Johnsen, S. & Iversen, B. B. (2006). *Phys. Rev. B*, **74**, 205105.

[bb6] Biswas, K., He, J., Blum, I. D., Wu, C.-I., Hogan, T. P., Seidman, D. N., Dravid, V. P. & Kanatzidis, M. G. (2012). *Nature*, **489**, 414–418. 10.1038/nature11439 22996556

[bb7] Božin, E. S., Malliakas, C. D., Souvatzis, P., Proffen, T., Spaldin, N. A., Kanatzidis, M. G. & Billinge, S. J. L. (2010). *Science*, **330**, 1660–1663. 10.1126/science.1192759 21164012

[bb8] Brown, D., Day, T., Borup, K., Christensen, S., Iversen, B. B. & Snyder, G. J. (2013*a*). *APL Mater.* **1**, 052107.

[bb9] Brown, D. R., Day, T., Caillat, T. & Snyder, G. J. (2013*b*). *J. Electron. Mater.* **42**, 2014–2019.

[bb10] Brown, D. R., Heijl, R., Borup, K. A., Iversen, B. B., Palmqvist, A. & Snyder, G. J. (2016). *Phys. Status Solidi RRL*, **10**, 618–621.

[bb11] Brown, D. R. & Snyder, G. J. (2016). *Innovative Thermoelectric Materials*, edited by H. E. Katz and T. O. Poehler, pp. 219–257. London: Imperial College Press.

[bb12] Caillat, T., Fleurial, J. P. & Borshchevsky, A. (1997). *J. Phys. Chem. Solids*, **58**, 1119–1125.

[bb13] Cargnoni, F., Nishibori, E., Rabiller, P., Bertini, L., Snyder, G. J., Christensen, M., Gatti, C. & Iversen, B. B. (2004). *Chem. Eur. J.* **10**, 3861–3870. 10.1002/chem.200400327 15317052

[bb14] Chi, H., Kim, H., Thomas, J. C., Shi, G. S., Sun, K., Abeykoon, M., Bozin, E. S., Shi, X. Y., Li, Q., Shi, X., Kioupakis, E., Van der Ven, A., Kaviany, M. & Uher, C. (2014). *Phys. Rev. B*, **89**, 195209.

[bb15] Christensen, S., Bindzus, N., Sist, M., Takata, M. & Iversen, B. B. (2016). *Phys. Chem. Chem. Phys.* **18**, 15874–15883. 10.1039/c6cp01730d 27240951

[bb16] Damay, F., Petit, S., Rols, S., Braendlein, M., Daou, R., Elkaïm, E., Fauth, F., Gascoin, F., Martin, C. & Maignan, A. (2016). *Sci. Rep.* **6**, 23415. 10.1038/srep23415 PMC4802330 27000414

[bb17] Danilkin, S. A., Avdeev, M., Sale, M. & Sakuma, T. (2012). *Solid State Ionics*, **225**, 190–193.

[bb18] Dolomanov, O. V., Bourhis, L. J., Gildea, R. J., Howard, J. A. K. & Puschmann, H. (2009). *J. Appl. Cryst.* **42**, 339–341.

[bb19] Frangis, N., Manolikas, C. & Amelinckx, S. (1991). *Phys. Status Solidi (A)*, **126**, 9–22.

[bb20] Gascoin, F. & Maignan, A. (2011). *Chem. Mater.* **23**, 2510–2513.

[bb21] Gulay, L., Daszkiewicz, M., Strok, O. & Pietraszko, A. (2011). *Chem. Met. Alloys*, **4**, 200–205.

[bb22] Heremans, J. P., Dresselhaus, M. S., Bell, L. E. & Morelli, D. T. (2013). *Nat. Nanotechnol.* **8**, 471–473. 10.1038/nnano.2013.129 23812187

[bb23] Heremans, J. P., Jovovic, V., Toberer, E. S., Saramat, A., Kurosaki, K., Charoenphakdee, A., Yamanaka, S. & Snyder, G. J. (2008). *Science*, **321**, 554–557. 10.1126/science.1159725 18653890

[bb24] Horvatić, M. & Vučić, Z. (1984). *Solid State Ionics*, **13**, 117–125.

[bb25] Kang, S. D., Danilkin, S. A., Aydemir, U., Avdeev, M., Studer, A. & Snyder, G. J. (2016). *New J. Phys.* **18**, 013024.

[bb26] Kashida, S. & Akai, J. (1988). *J. Phys. C. Solid State Phys.* **21**, 5329–5336.

[bb27] Liu, H., Shi, X., Kirkham, M., Wang, H., Li, Q., Uher, C., Zhang, W. & Chen, L. (2013*a*). *Mater. Lett.* **93**, 121–124.

[bb28] Liu, H., Shi, X., Xu, F., Zhang, L., Zhang, W., Chen, L., Li, Q., Uher, C., Day, T. & Snyder, G. J. (2012). *Nat. Mater.* **11**, 422–425. 10.1038/nmat3273 22406814

[bb29] Liu, H., Yuan, X., Lu, P., Shi, X., Xu, F. F., He, Y., Tang, Y. S., Bai, S. Q., Zhang, W. Q., Chen, L. D., Lin, Y., Shi, L., Lin, H., Gao, X. Y., Zhang, X. M., Chi, H. & Uher, C. (2013*b*). *Adv. Mater.* **25**, 6607–6612. 10.1002/adma.201302660 24018747

[bb30] Lu, P., Liu, H., Yuan, X., Xu, F., Shi, X., Zhao, K., Qiu, W., Zhang, W. & Chen, L. (2015). *J. Mater. Chem. A*, **3**, 6901–6908.

[bb31] Lutterotti, L. (2010). *Nucl. Instrum. Methods Phys. Res. B*, **268**, 334–340.

[bb32] Mahan, G. D. (2015). *J. Appl. Phys.* **117**, 045101.

[bb33] Milat, O., Vučić, Z. & Ruščić, B. (1987). *Solid State Ionics*, **23**, 37–47.

[bb34] Miyatani, S. (1973). *J. Phys. Soc. Jpn*, **34**, 423–432.

[bb35] Müller, U. (2013). *Symmetry Relationships Between Crystal Structures*, pp. 197–215. Oxford University Press.

[bb36] Nguyen, M. C., Choi, J.-H., Zhao, X., Wang, C.-Z., Zhang, Z. & Ho, K.-M. (2013). *Phys. Rev. Lett.* **111**, 165502. 10.1103/PhysRevLett.111.165502 24182279

[bb37] Pei, Y., LaLonde, A., Iwanaga, S. & Snyder, G. J. (2011). *Energ. Environ. Sci.* **4**, 2085–2089.

[bb38] Peijie, S., Niels, O., Simon, J., Bo, B. I. & Frank, S. (2009). *Appl. Phys. Expr.* **2**, 091102.

[bb39] Popa, N. C. (1998). *J. Appl. Cryst.* **31**, 176–180.

[bb40] Rahlfs, P. (1936). *Z. Phys. Chem. B*, **31**, 157–194.

[bb41] Sheldrick, G. M. (2015). *Acta Cryst.* A**71**, 3–8.

[bb42] Sirusi, A. A., Ballikaya, S., Uher, C. & Ross, J. (2015). *J. Phys. Chem. C*, **119**, 20293–20298.

[bb43] Snyder, G. J., Christensen, M., Nishibori, E., Caillat, T. & Iversen, B. B. (2004). *Nat. Mater.* **3**, 458–463. 10.1038/nmat1154 15220913

[bb44] Snyder, G. J. & Toberer, E. S. (2008). *Nat. Mater.* **7**, 105–114. 10.1038/nmat2090 18219332

[bb45] Søndergaard, M., Johnsen, S., Sun, P., Sun, Y., Cenedese, S., Gatti, C., Steglich, F. & Iversen, B. (2013). *Thermoelectric Nanomaterials*, edited by K. Koumoto and T. Mori, pp. 71–93. Heidelberg, Berlin: Springer.

[bb46] Sun, P., Wei, B., Zhang, J., Tomczak, J. M., Strydom, A. M., Søndergaard, M., Iversen, B. B. & Steglich, F. (2015). *Nat. Commun.* **6**, 7475. 10.1038/ncomms8475 PMC4491185 26108283

[bb47] Takahashi, T., Yamamoto, O., Matsuyama, F. & Noda, Y. (1976). *J. Solid State Chem.* **16**, 35–39.

[bb48] Van Der Lee, A. & Wiegers, G. A. (1989). *J. Solid State Chem.* **82**, 216–224.

[bb49] Vučić, Z., Horvatić, V. & Milat, O. (1984). *Solid State Ionics*, **13**, 127–133.

[bb50] Vučić, Z., Horvatić, V. & Ogorelec, Z. (1982). *J. Phys. C Solid State Phys.* **15**, 3539–3546.

[bb51] Vučić, Z., Milat, O., Horvatić, V. & Ogorelec, Z. (1981). *Phys. Rev. B*, **24**, 5398–5401.

[bb52] Xcalibur, C. (2010). *Xcalibur/CCD System, CrysAlis Pro Software System.* Version 2010, 1. Agilent Technologies UK Ltd, UK.

[bb53] Yamamoto, K. & Kashida, S. (1991). *Solid State Ionics*, **48**, 241–248.

[bb54] Yang, L., Chen, Z.-G., Han, G., Hong, M., Zou, Y. & Zou, J. (2015). *Nano Energy*, **16**, 367–374.

[bb55] Yu, B., Liu, W., Chen, S., Wang, H., Wang, H., Chen, G. & Ren, Z. (2012). *Nano Energy* **1**, 472–478.

[bb56] Zeier, W. G., Zevalkink, A., Gibbs, Z. M., Hautier, G., Kanatzidis, M. G. & Snyder, G. J. (2016). *Angew. Chem. Int. Ed.* **55**, 6826–6841. 10.1002/anie.201508381 27111867

[bb57] Zhang, X. & Zhao, L.-D. (2015). *J. Materiomics*, **1**, 92–105.

[bb58] Zhao, L. L., Wang, X. L., Wang, J. Y., Cheng, Z. X., Dou, S. X., Wang, J. & Liu, L. Q. (2015*a*). *Sci. Rep.* **5**, 7671. 10.1038/srep07671 PMC5378988 25567317

[bb59] Zhao, L. L., Wang, X. L., Yun, F. F., Wang, J. Y., Cheng, Z. X., Dou, S. X., Wang, J. & Snyder, G. J. (2015*b*). *Adv. Electron. Mater.* **1**, 1400.

