# Supplementary material for: Crystal structure across the β to α phase transition in thermoelectric Cu2−xSe
Source: IUCrJ. 2017 Jun 13;4(Pt 4):476–85. doi: 10.1107/S2052252517005553 (PMC5571810; doi:10.1107/S2052252517005553)
Supplement: Supplementary file 11 [file m-04-00476-sup12.pdf]

IUCrJ

Volume 4 (2017)

Supporting information for article:

Crystal structure across the β to α phase transition in thermoelectric Cu2−*x*Se

Espen Eikeland, Anders B. Blichfeld, Kasper A. Borup, Kunpeng Zhao, Jacob Overgaard, Xun Shi, Lidong Chen and Bo B. Iversen

S1. Additional SCXRD measurements

In addition to analyzing diffraction data from single crystals synthesized using the chemical vapor transport (CVT) method, single crystal X-ray diffraction data have also been measured on a smaller single crystal with dimensions 0.07 x 0.03 x 0.01 mm^3 taken from a synthesized bulk sample.

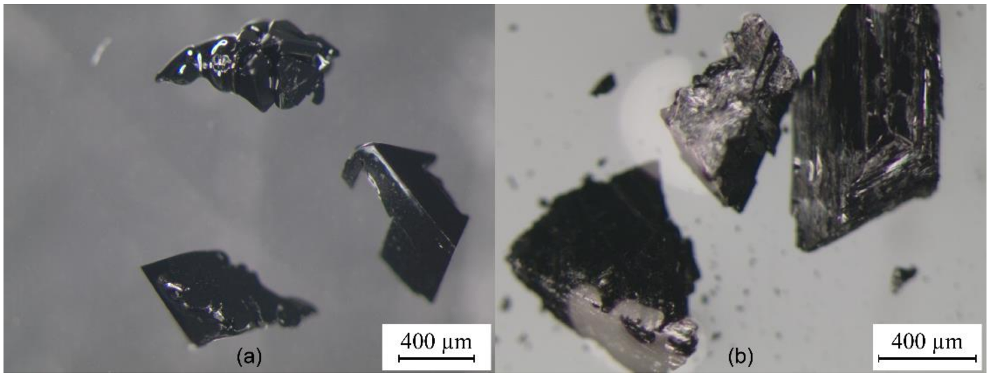

Figure S1 Micrographs of $Cu_{2-X}Se$; bulk sample (a), x ~ 0, and CVT sample (b), x ~ 0.05(13).

Pictures of the two samples are shown above, with crystallographic information listed below. The corresponding CIF's are labelled ***Cu_{2-X}Se_sample_temperature.*** CVT1 & 2 refer to two different single crystals from the same sample, where the CVT2 sample had been heated to 450 K before collecting diffraction data at 100K.

Table S1 Selected crystallographic information

Sample*	**bulk**	**CVT2**	**CVT1**
**Chemical formula**	$Cu_{1.89(1)}Se$	$Cu_{1.77(3)}Se$	$Cu_{1.95}Se$
**M_r / g · mol^{-1}**	199	196	202
**T / K**	100	100	295
**Crystal system**	trigonal	trigonal	trigonal
**Space group**	$R\bar{3}m$; H	$R\bar{3}m$; H	$R\bar{3}m$; H
**a / Å**	4.102(2)	4.102(1)	4.1227(8)
**c / Å**	20.533(9)	20.420(7)	20.449(6)
**Volume / Å^3**	299.2(3)	297.5(2)	301.0(2)
**Z**	6	6	6
**ρ_{calc} / g·cm^{-3}**	6.625	6.375	6.711
**μ / mm^{-1}**	37.884	36.688	38.277
**F(000)**	533	524	543
**Crystal size / mm^3**	0.07 × 0.025 × 0.015	0.11 × 0.09 × 0.03	0.017 × 0.05 × 0.09
**$(\sin\theta/\lambda)_{max}$ / Å^{-1}**	0.55	0.62	0.62
**$N_{Tot,obs}$**	785	667	809
**$N_{Uniq,obs}$**	72	98	102
**$N_{Parameters}$**	15	17	19
**GOF**	1.07	1.249	1.210
**R_{int}**	0.14	0.074	0.075
**R_1 , $R_1[F^2>2\sigma(F^2)]$**	0.086, 0.079	0.0602, 0.060	0.061, 0.059
**wR_2, $wR_2[F^2>2\sigma(F^2)]$**	0.192, 0.184	0.167, 0.167	0.158, 0.156
**$\Delta\rho_{max}$, $\Delta\rho_{min}$ / e Å^{-3}**	2.74, -1.28	1.81, -2.71	1.33, -1.39

S2. TEM – EDX

To investigate the composition of the single crystals made by CVT, energy dispersive X-ray spectrometry (EDX) was performed. The crystal was crushed in an agate mortar and dispersed in ethanol. The dispersion was applied to a Si_4N_3 TEM-grid, to avoid signal from a normal Cu-grid. A Talos F220A from FEI operating at 200 kV, with a TWIN lens system, X-FEG electron source, Ceta 16M Camera and a Super-X EDX Detector was used. Spatially resolved EDX analysis, with a spatial resolution better than 2 nm, was obtained

using the microscope in STEM mode. Scanning Transmission Electron Microscopy and EDX was performed by Mohammad Aref Hasen Mamakhel. The composition was $Cu_{1.95(13)}Se$.

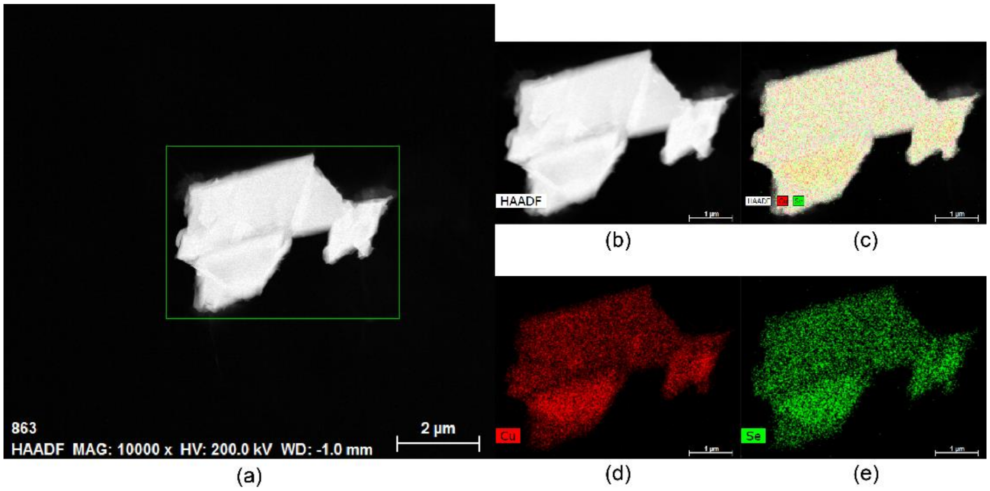

Figure S2 Elemental composition of the CVT sample.

Bruker Nano GmbH, Germany				01/03/2016
	Quantax			
Results	Map			
Date:	01/03/2016			
Element	[norm. wt.%]	$std_{3\ sigma}$ wt.%	[norm. at.%]	$std_{3\ sigma}$ at.%
Copper	61.03	5.64	66.05	6.10
Selenium	38.97	3.70	33.95	3.22
Sum:	100		100	

Bruker Nano GmbH, Germany				01/03/2016
	Quantax			
Results	Map			
Date:	01/03/2016			
Element	[norm. wt.%]	$std_{3\ sigma}$ wt.%	[norm. at.%]	$std_{3\ sigma}$ at.%
Copper	61.01	5.64	66.04	6.10
Selenium	38.99	3.70	33.96	3.22
Sum:	100		100	

STEM on Cu_2Se (99.95 % Sigma ALDRICH: 481629) on Si_4N_3 grid to avoid any signal from a normal copper grid: $Cu_{1.99(6)}Se$

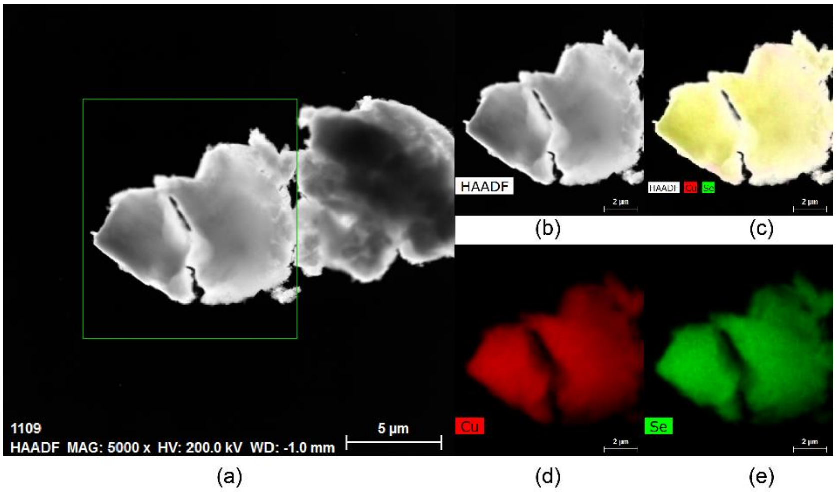

Figure S3 Elemental composition of the standard.

Bruker Nano GmbH, Germany				02/09/2016
	Quantax			
Results	Map			
Date:	02/09/2016			
Element	[norm. wt.%]	std$_{3\ sigma}$ wt.%	[norm. at.%]	std$_{3\ sigma}$ at.%
Copper	61.66	66.65	3.83	4.14
Selenium	38.34	33.35	2.32	2.02
Sum:	100		100	

Bruker Nano GmbH, Germany				02/09/2016
	Quantax			
Results	Map			
Date:	02/09/2016			
Element	[norm. wt.%]	std$_{3\ sigma}$ wt.%	[norm. at.%]	std$_{3\ sigma}$ at.%
Copper	61.66	66.65	3.83	4.14
Selenium	38.34	33.35	2.32	2.02
Sum:	100		100	

Bruker Nano GmbH, Germany				02/09/2016
	Quantax			
Results	Map			
Date:	02/09/2016			
Element	[norm. wt.%]	std$_{3\ sigma}$ wt.%	[norm. at.%]	std$_{3\ sigma}$ at.%

Copper	61.66	66.65	3.83	4.14
Selenium	38.34	33.35	2.32	2.02
Sum:	100		100	

Bruker Nano GmbH, Germany				02/09/2016
	Quantax			
Results	Map			
Date:	02/09/2016			
Element	[norm. wt.%]	std$_{3\ \mathrm{sigma}}$ wt.%	[norm. at.%]	std$_{3\ \mathrm{sigma}}$ at.%
Copper	61.66	66.65	3.83	4.14
Selenium	38.34	33.35	2.32	2.02
Sum:	100		100	

S3. Data Integration, determining unit cell and solving the average structure

In the following the steps in analyzing the collected SCXRD data are elaborated. The data collected at 295 K are chosen as an example since the data obtained at 100 K are slightly twinned complicating the procedure, as seen in Figure S4.

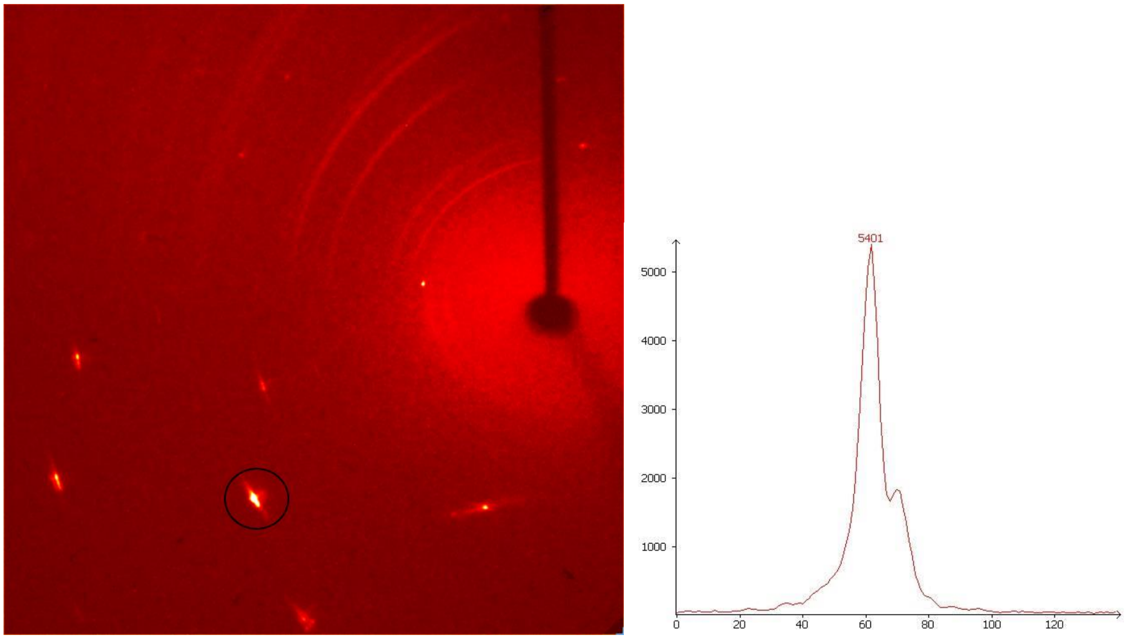

Figure S4 Selected diffraction frame from the data collected at 100 K showing a twinned peak. Insert show the intensity of the encircled peak.

Figure S5

S3.1. Determining the unit cell.

98.85 % of peaks with intensities (I) > 10.000 counts can be indexed using the following reduced unit cell; *a* = 4.1282 Å, *b* = 4.1317 Å, *c* = 7.2185 Å, α = 73.5897°, β = 73.6649°, γ= 60.0971° and V = 100.9 Å^3.

By applying simple transformation matrices, the relevant monoclinic and trigonal unit cells can be constructed. The smallest monoclinic cell using the transformation matrix $\boldsymbol{T}_{\text{monoclinic}} = \begin{pmatrix} 1 & 1 & 0 \\ 1 & -1 & 0 \\ 0 & 0 & -1 \end{pmatrix}$ gives the following unit cell parameters: *a* = 7.152(3) Å, *b* = 4.1387(15) Å, *c* = 7.226(4) Å, α = 89.95(4)°, β = 108.94(4)°, γ = 90.02(3)° and V = 202.28(15) Å^3. The reciprocal space including this monoclinic cell together with all peaks I > 1000 are depicted below.

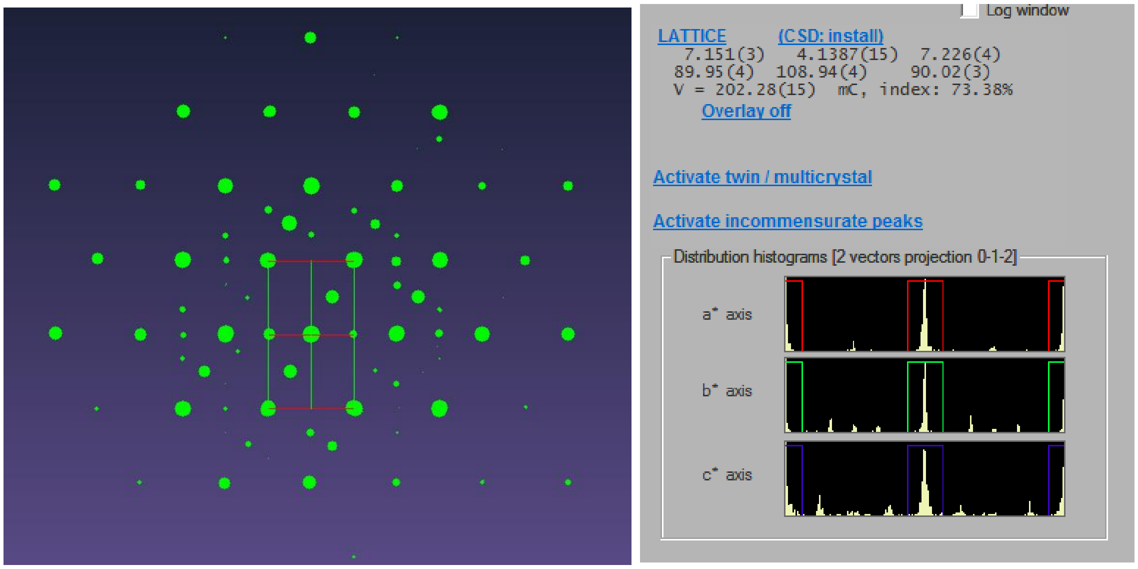

A significant number of peaks are located at different fractions along the reciprical unit cell axes, which can be used to construct almost all the suggested monoclinic unit cells in the litterature.

$\boldsymbol{T}_{\text{Milat(1987)}} = \begin{pmatrix} 1 & 0 & 0 \\ 0 & 3 & 0 \\ 0 & 0 & 0 \end{pmatrix}$ → : *a* = 7.152(3) Å, *b* = 12.418(15) Å, *c* = 7.226(4) Å, α = 89.95(4)°, β = 108.94(4)°, γ = 90.02(3)° and V = 607.0(5) Å^3 (Milat *et al.*, 1987, Lu *et al.*, 2015)

$\boldsymbol{T}_{\text{Gulay(2011)}} = \begin{pmatrix} 1 & 0 & 0 \\ 0 & 3 & 0 \\ 1 & 0 & 4 \end{pmatrix}$ → : *a* = 7.1535(16) Å, *b* = 12.415(3) Å, *c* = 27.440(7) Å, α = 90.039(19)°, β = 94.694(19)°, γ = 90.011(18)° and V = 2428.9(10) Å^3 (Gulay *et al.*, 2011)

The transformation matrix $\boldsymbol{T}_{\text{trigonal}} = \begin{pmatrix} 1 & 0 & 0 \\ -1 & 1 & 0 \\ -1 & -1 & 3 \end{pmatrix}$ constructs a trigonal unit cell (using the hexagonal unit cell setting) from the reduced cell giving the cell parameters: *a* = 4.129(2) Å, *b* = 4.1397(15) Å, *c* = 20.50(1) Å, α = 90.02(3)°, β = 90.20(4)°, γ = 120.07(4)° and V = 303.3(2) Å^3. Ceciprocal space including this unit cell together with all peaks Int > 1000 are depicted below. Notice the same orientation as the figure above. Again a significant number of peaks are located at different fractions along the reciprical unit cell axes, which can be used to construct the suggested trigonal cells from the literature using simple transformation matrices.

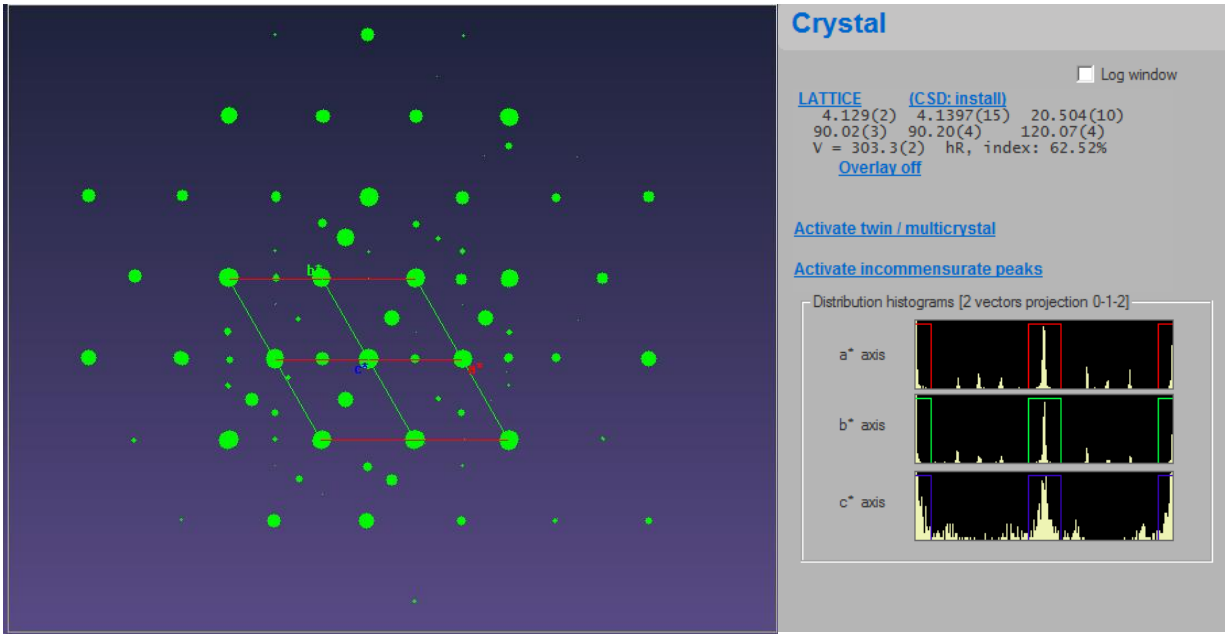

$\boldsymbol{T}_{\text{Vučić(1981)}} = \begin{pmatrix} 3 & 0 & 0 \\ 0 & 3 & 0 \\ 0 & 0 & 2 \end{pmatrix}$ → : *a* = 12.(16) Å, *b* = 12.415(3) Å, *c* = 27.440(7) Å, α = 90.039(19)°, β = 94.694(19)°, γ = 90.011(18)° and V = 2428.9(10) Å^3 (Vučić *et al.*, 1981)

S3.2. Data integration:

Table S2 shows the results of 4 selected integrations using different unit cell parameters and space-group symmetries. The integrations have been selected due to their relatively low R_{int} and are in the following discussed in the context of possible structure solutions. The resulting structural models are not based on any model from the literature.

Table S2 Integration parameters for a selected number of integrations and the resulting R1 factor for the refined models.

**Space group**	*a (Å), b(Å), c (Å),* α(°)*,* β (°), γ (°), V *(Å*^{3}*)*	R_{int}	I/sig	Competeness (%) (resolution (sin θ/λ)$_{max}$ = 0.8 Å^{-1})	R1 (%)
$\boldsymbol{R\bar{3}m}$**; H (1)**	4.137(3), 4.137(3), 20.46(3), 90, 90, 120, 303.3(5)	7.4	22.9	100	5.7
**C2/c (2)**	7.1477(9), 4.1263(3), 7.2022(9), 90, 108.88(1) 90, 200.99(4)	4.0	22.1	100	5.6
**P2$_1$/m (3)**	7.203(1), 4.1270(5), 7.152(1), 90. 108.85(2) 90, 201.19(6)	5.8	9.4	100	8.3
**C2/c (1x3x4 cell) (4)**	7.152(1), 12.384(1), 28.817(7), 90, 108.84(2) 90, 2415.8(9)	8.5	4.3	100	11.9

The diffraction images were first integrated without a lattice extinction filter and outlier rejection. For the background evaluation the "smart background" was applied with frame range 1, evaluation range 15 and repeat frequency 15. During the absorption correction the outlier rejection was used and the Fridel pairs set to be equivalent. The applied absorption was the automated empirical absorption correction.

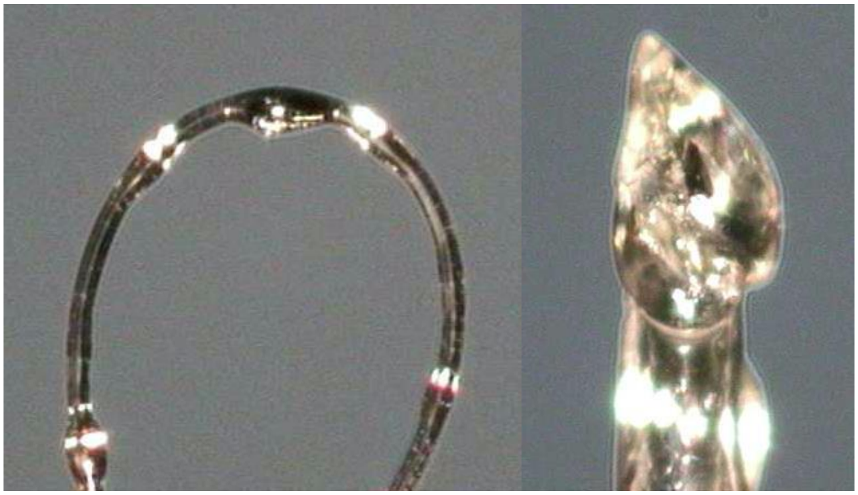

Figure S6 Image of the same crystal mounted on a loop using paratone oil (left), for the LT 100 K and 295 K experiments, and mounted on an amorphous glass rod in Epoxy, two component glue, (right) for the HT experiments.

S3.3. Structure solutions

Structure solutions giving acceptable refinement parameters are found for the two first entries in Table S2 (**1, 2**) including all main reflections. It should be noted that the two refinements integrate precicely the same reflections. The resulting structural arrangements are also identical, with the resulting R1 factor being the same. Since solution **1** have a higher symmetry (trigonal vs monoclinic) we have choosen to continue with this structure. For the structural model, the only constraint is on the total Cu occupancy, which is fixed to the stochiometry found by the elemental analysis. Solution **2** has a lower R_{int}, but this is likely just a result of the lower symmetry. Slices of reciprocal space for **1** are shown below (100 K) illustrating the number of peaks not indexed.

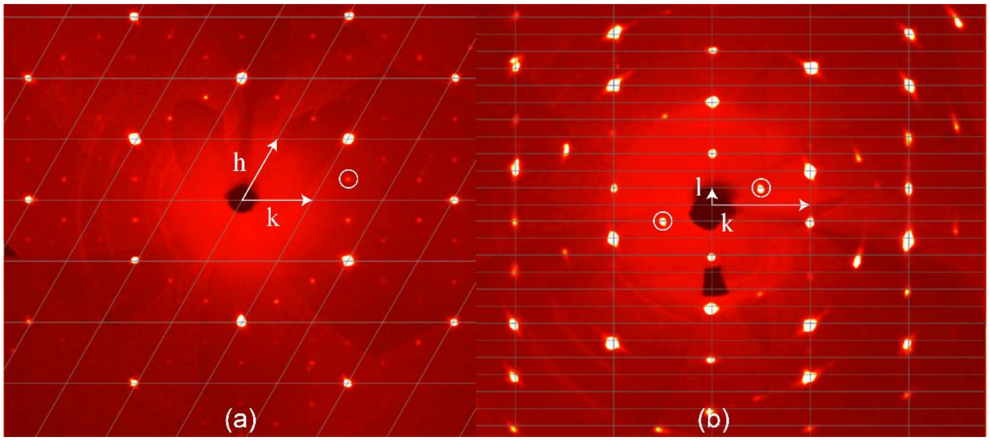

Figure S7 Slices of reciprocal space (100 K) showing the (hk0) plane, (a), and the (0kl) plane, (b). Important non-indexed peaks are marked by a white circle.

Lattice exceptions:	P	A	B	C	I	F	Obv	Rev	All
N (total) =	0	1191	1199	1178	1201	1784	1599	1593	2382
N (int>3sigma) =	0	503	630	133	585	633	759	779	1144
Mean intensity =	0.0	34.2	36.0	2.0	62.7	24.2	53.9	61.8	58.8
Mean int/sigma =	0.0	5.2	6.4	1.3	7.7	4.3	7.1	7.8	7.4

Figure S8 Lattice exceptions.

In Figure S6b the encircled reflections are not indexed using the unit cell from solution **1** and **2**, but are included using solution **3** which have the same monoclinic unit cell parameters as **2** but no C-centered. The extra peaks in Figure S6a are not included in **3**. As seen from Figure S8 below the reflections breaking the C-centering are very weak. A satisfactory structural model could not be obtained from integration **3**.

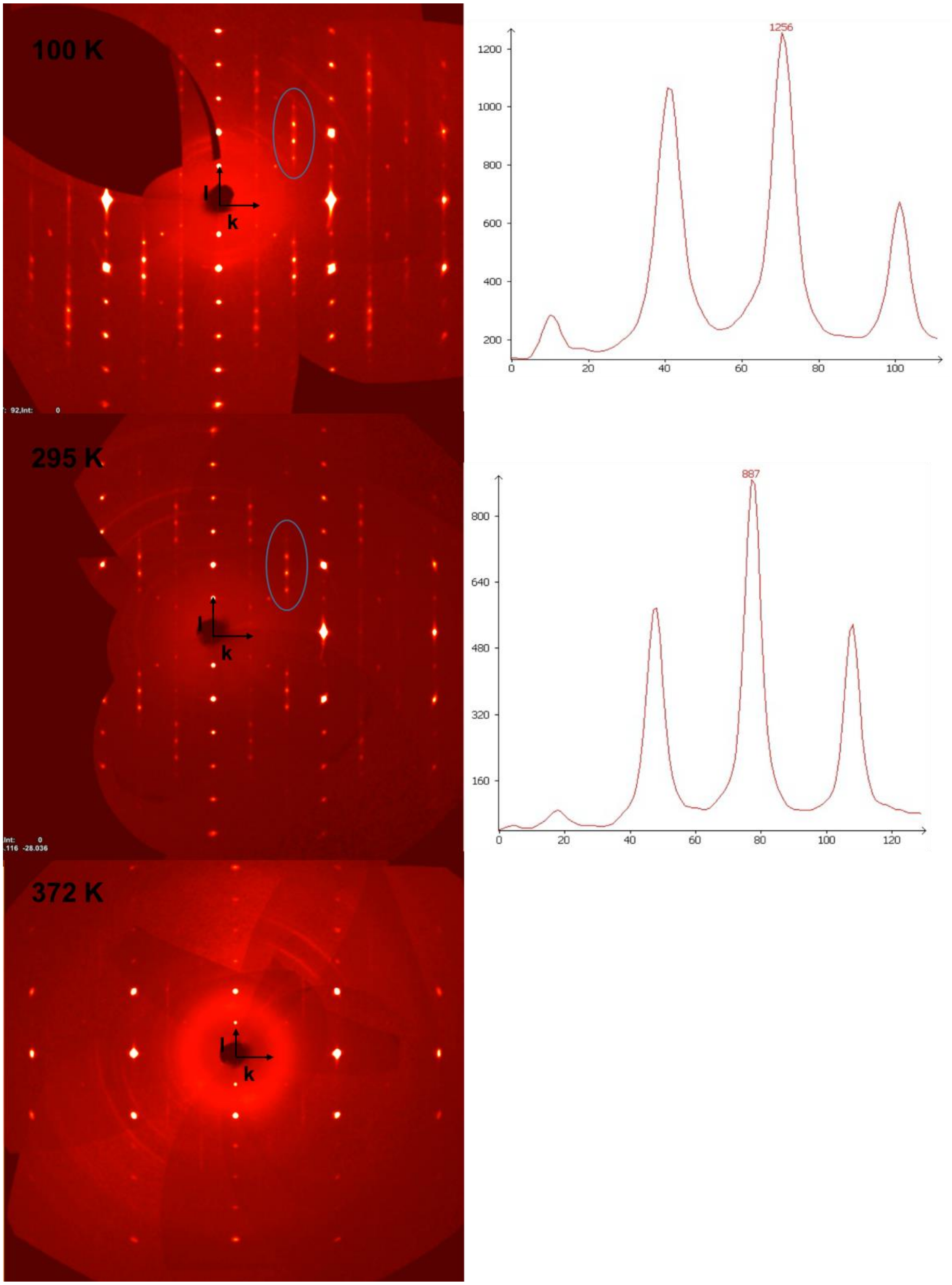

Figure S9 Slices of reciprocal space ((0kl) using the monoclinic integration **2**,**3** or **4**) for the diffraction data obtained at 100 K, 295 K, and 372 K using an exposure time of 140 s, 60 s and 70 s, respectively. The contrast is set to **1k** in all three diffractograms. The 1x3x4 supercell (**4**) indexes all the shown reflections at

100 K, while all reflections at 372 K can be indexed using the small monoclinic cell **3**. Inserts show the peak size of the 4 peaks marked using blue circles. The weak powder rings originate from the instrument and not the sample.

Using integration **4** (integrating the same reflections as the unit cell suggested by Gulay *et al.* (2011)), which is a 1x3x4 super cell of the unit cell in integration **2**, integrates the main part of all weak super-structure reflections (by removing the C centering additional peaks can be included in the integration) A structural model is obtainable from the integration with a resulting **R1** value of11.8 % at 295 K. From Figure S8 it can be seen that the superstructure reflections are dissapearing at 372 K. The intensity of the main peaks in the figure at 295 K and 372 K have comparable intensities. It should also be noted that the along the *c*-axis there seems to be some indications of diffuse scattering.

From Figure S6a, if we go back to the trigonal unit cell, it looks like a trigonal 3x3x1 supercell could index all the reflections in the figure. However, this is the result of the diffuse scattering oriented along the *c*-axis. The super-structure peaks do not have maxima in the depicted layer.

In summary, the symmetry of the main reflections is trigonal, while it is likely that the symmetry of the complete structure including superstructure peaks are not trigonal. In addition there seems to be diffuse scattering along the *c*-axis. The diffuse scattering along the *c*-axis explains why there are so many unassigned peaks along this direction in the reciprocal space after peak indexing (from the peak hunting).

S3.4. Possible super-structure models

The Gulay model (Gulay *et al.*, 2011):

In 2011 Gulay *et al*. studied $Cu_{2-x}Se$ using SCXRD and PXRD. They collected and integrated SCXRD data using space group C2/c and unit cell parameters a = 7.1379(4) Å, b = 12.3823(7) Å, c = 27.3904(9) Å, β = 94.308º. The unit cell integrates the same reflections as integration **4** (a = 7.152(1) Å, b = 12.384(1) Å, c = 28.817(7), β = 108.84(2)º) with the following transformation matrix relating the two unit cells:

$$\mathbf{T}_{\mathrm{Gulay},\mathbf{4}} = \begin{pmatrix} 1 & 0 & 0 \\ 0 & 1 & 0 \\ -1 & 0 & 1 \end{pmatrix}$$

The only information Gulay *et al*. writes concerning their structural model from SCXRD is the following:

"*A model of the structure [LT-Cu_{2-x}Se] was obtained from X-ray single crystal diffraction data (R1 ≈ 0.14) at room temperature. At the second step, X-ray powder diffraction data were used for the refinement*"

Since the authors were not satisfied with the high **R1** value of 0.14, indicating that their structural model do not fit their diffraction data, they instead used the model on their PXRD data resulting in a **R1** value of 0.0765.

It should be stated here that this procedure of using an incorrect model based on SCXRD to fit PXRD data is not considered acceptable. Obviously a correct structural model has to be able to describe the SCXRD data.

That being said it is informative to see how their proposed model fits our collected SCXRD data. Unfortunately, they only publish their structural model after it has been refined against their powder diffraction data. The comparison is therefore based on this structure. The $\mathbf{T}_{\mathrm{Gulay},\mathbf{4}}$ matrix has been used in order to get the correct fractional coordinates for the reflection list from integration **4.**

Table S3 The Gulay model taken from (Gulay et al., 2011) with fractional coordinates recalculated for the unit cell *a* = 7.152(9) Å, *b* = 12.384(1) Å, *c* = 28.821(5), *β* = 108.87(1)º

Atom	x/*a*	y/*b*	z/*c*	Uequivalient (Å^2)
**Cu01**	-0.0662	0.3250	0.3198	0.025
**Cu02**	0.0684	0.6650	0.4284	0.025
**Cu03**	-0.0469	-0.0050	0.3221	0.025
**Cu04**	0.1112	0.3270	0.4562	0.025
**Cu05**	0.3742	-0.0450	0.3982	0.025
**Cu06**	0.5823	0.4910	0.4293	0.025
**Cu07**	0.7244	0.6550	0.3514	0.025
**Cu08**	-0.0964	0.6610	0.2886	0.025
**Cu09**	0.1327	0.5250	0.3567	0.025
**Cu10**	0.2958	0.3390	0.3918	0.025
**Cu11**	0.5962	0.3030	0.3552	0.025
**Cu12**	0.4110	0.6860	0.3980	0.025
**Se01**	0.2518	-0.1730	0.4448	0.013
**Se02**	0.2477	0.3330	0.3037	0.013
**Se03**	0.2614	-0.0130	0.3104	0.013
**Se04**	0.2765	0.4970	0.4445	0.013
**Se05**	-0.2381	0.6520	0.4409	0.013
**Se06**	-0.2438	0.1620	0.3082	0.013

Table S4 The Gulay model used to fit the SCXRD data collected in this work, also at room temperature (295 K). a = 7.152(1) Å, b = 12.384(1) Å, c = 28.817(7), β = 108.84(2)°, space group C2/c.

Refined parameters	Scale	Scale+ADP*	Scale+ADP*+xyz
**All reflections**			
$N_{Uniq,obs}$	2462	2462	2462
$N_{Parameters}$	1	3	57
GOF	3.272	3.141	2.016
R_1, $R_1[F^2>2\sigma(F^2)]$	0.600, 0.533	0.574, 0.506	0.363, 0.294
wR_2, $wR_2[F^2>2\sigma(F^2)]$	0.849, 0.795	0.836, 0.775	0.656, 0.606
$\Delta\rho_{max}$, $\Delta\rho_{min}$ / e Å^{-3}	115.9, -13.60	92.51, -11.40	31.43, -6.44
**Main reflections only**			
$N_{Uniq,obs}$	236	236	236
$N_{Parameters}$	1	3	57
GOF	1.508	1.415	2.538
R_1, $R_1[F^2>2\sigma(F^2)]$	0.610, 0.609	0.557, 0.555	0.275, 0.272
wR_2	0.654	0.587	0.510
$\Delta\rho_{max}$, $\Delta\rho_{min}$ / e Å^{-3}	33.68, -4.17	21.01, -4.74	11.64, -4.31
**Super-structure reflections only**			
$N_{Uniq,obs}$	2226	2226	2226
$N_{Parameters}$	1	3	57
GOF	3.095	3.212	2.220
R_1, $R_1[F^2>2\sigma(F^2)]$	0.570, 0.411	0.573, 0.423	0.476, 0.356
wR_2	0.829	0.837	0.7184
$\Delta\rho_{max}$, $\Delta\rho_{min}$ / e Å^{-3}	13.13, -10.67	10.69, -8.79	4.64, -4.50

*All Cu atoms are constrained to having equivalent ADPs. All Se atoms are constrained to having equivalent ADPs

In the table above refinement intecators are listed showing how well the Gulay model fits the SCXRD data presented in this work. Three models have been used: one where only the scale factor have been refined, one where additional isotropic ADPs for each element were refined, and on where the atom coordinates were also refined. Furthermore, the list of reflections have been divided into main and super-structure reflections in order to see how the individual models fit the main and super-structure peaks, respectively.

From the tabulated data it is evident that the Gulay model does not fit our SCXRD data, neither the main reflections nor the super-structure peaks. Even when freely refining all atomic positions the overall **R1** value is still 0.294. It should be noted that the refinements are all stable, but there is a lot of electron density

unaccounted for. Below is a electron difference map, with a contour of 20 e$Å^{-3}$, for the model with atomic coordinates as free parameters. Clear indicators of disordered Cu are observed.

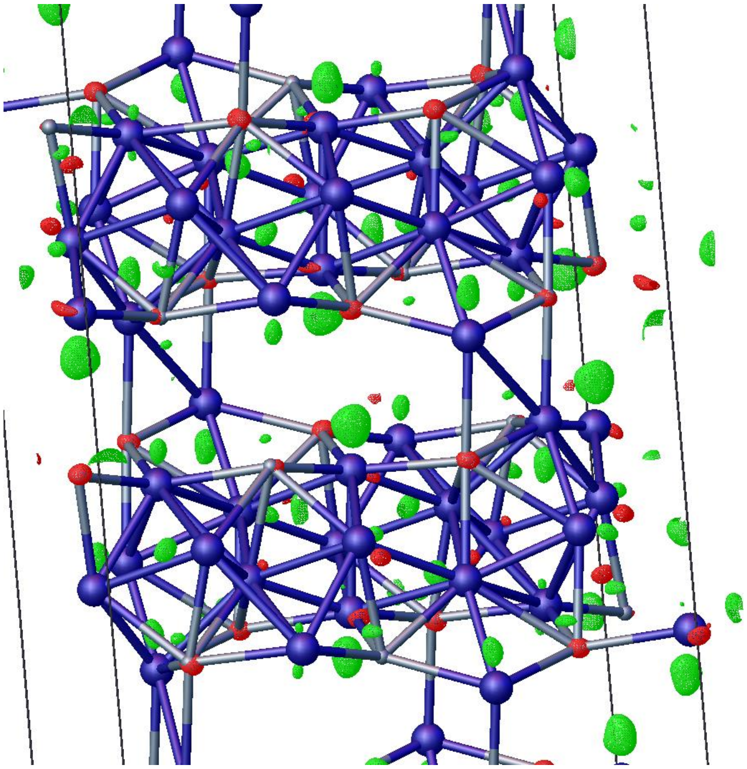

Figure S10 Electron difference map, with a contour of 20 e$Å^{-3}$. The green surfaces show areas of electron density missing from the model, and red surfaces indicate the opposite. The structural model used is the Gulay model with refined scale, ADP and xyz parameters, fit to all reflections (main + superstructure reflections). Cu (blue), Se (grey).

In summary, it is evident that the structural model proposed by Gulay *et al*. (2011) does not fit their own original SCXRD data (**R1** ≈ 0.14**)** nor does itfit the SCXRD data presented here (**R1 =** 0.294).

S3.5. Ordered super-structure

If the suggested ordering in section 6 (main paper) is long-range order then a possible super-structure model can be constructed. The ordering is depicted in Figure 5*b* & *d* and the fractional coordinates are given below.

Table S5 Coordinates for super-structure bases on proposed Cu ordering (295 K), *a* = 7.152(1) Å, *b* = 12.384(1) Å, *c* = 28.817(7), *β* = 108.84(2)º, space group C2/c.

**Atom**	**x/*a***	**y/*b***	**z/*c***
**Se1**	0.2410	0.3333	0.6808
**Se2**	0.2410	0	0.6808
**Se3**	0.2590	0.1667	0.8192
**Se5**	0.2410	0	0.9308
**Se6**	0.2410	0.3333	0.9310
**Se4**	0.7410	0.1667	0.9308
**Cu1**	0.3856	0.1667	0.9141
**Cu2**	0.9324	0.3333	0.9493
**Cu3**	0.9324	0	0.9493
**Cu4**	0.2097	0.1667	0.9808
**Cu5**	0.8934	0.2010	0.0192
**Cu6**	0.6068	0.0344	0.9808
**Cu7**	0.3856	0.1667	0.6641
**Cu8**	0.0676	0.3333	0.8007
**Cu9**	0.0676	0	0.8007
**Cu10**	0.2097	0.1667	0.7308
**Cu11**	0.3934	0.2990	0.7692
**Cu12**	0.3932	0.0344	0.7692

Table S6 The ordered model used to fit the SCXRD data collected in this work, at room temperature (295 K). *a* = 7.152(1) Å, *b* = 12.384(1) Å, *c* = 28.817(7), *β* = 108.84(2)°, space group C2/c.

Refined parameters	Scale+ADP*	Scale+ADP*+xyz
**All reflections**		
$N_{Uniq,obs}$	2462	2462
$N_{Parameters}$	2	58
GOF	3.213	2.617
R_1, $R_1[F^2>2\sigma(F^2)]$	0.592, 0.538	0.445, 0.378
wR_2, $wR_2[F^2>2\sigma(F^2)]$	0.872, 0.845	0.776, 0.726
$\Delta\rho_{max}$, $\Delta\rho_{min}$ / e Å^{-3}	48.96, -7.64	20.33, -13.52
**Main reflections only**		
$N_{Uniq,obs}$	236	236
$N_{Parameters}$	2	58
GOF	2.146	4.331
R_1, $R_1[F^2>2\sigma(F^2)]$	0.403, 0.399	0.287, 0.283
wR_2	0.587	0.686
$\Delta\rho_{max}$, $\Delta\rho_{min}$ / e Å^{-3}	13.34, -4.03	16.71, -10.01
**Super-structure reflections only**		
$N_{Uniq,obs}$	2226	2226
$N_{Parameters}$	2	58
GOF	3.769	2.601
R_1, $R_1[F^2>2\sigma(F^2)]$	0.725, 0.648	0.582, 0.476
wR_2	0.919	0.816
$\Delta\rho_{max}$, $\Delta\rho_{min}$ / e Å^{-3}	9.61, -9.30	8.56, -6.58

Table S6 above lists refinement indicator for how an ordered model fits the SCXRD data (295 K). The ordered structural model does not describe the diffraction data (R1 = 0.378), neither the main reflections (R1 = 0.283) nor the super-structure reflections (R1 = 0.476). Below in Figure S10 an electron difference map is shown, with a contour of 20 eÅ-3, for the model with refined ADPs and atomic positions. The difference map show that the Cu sites contain too much electron density (red) while there is also a lot of disordered Cu sites not described by the model (green).

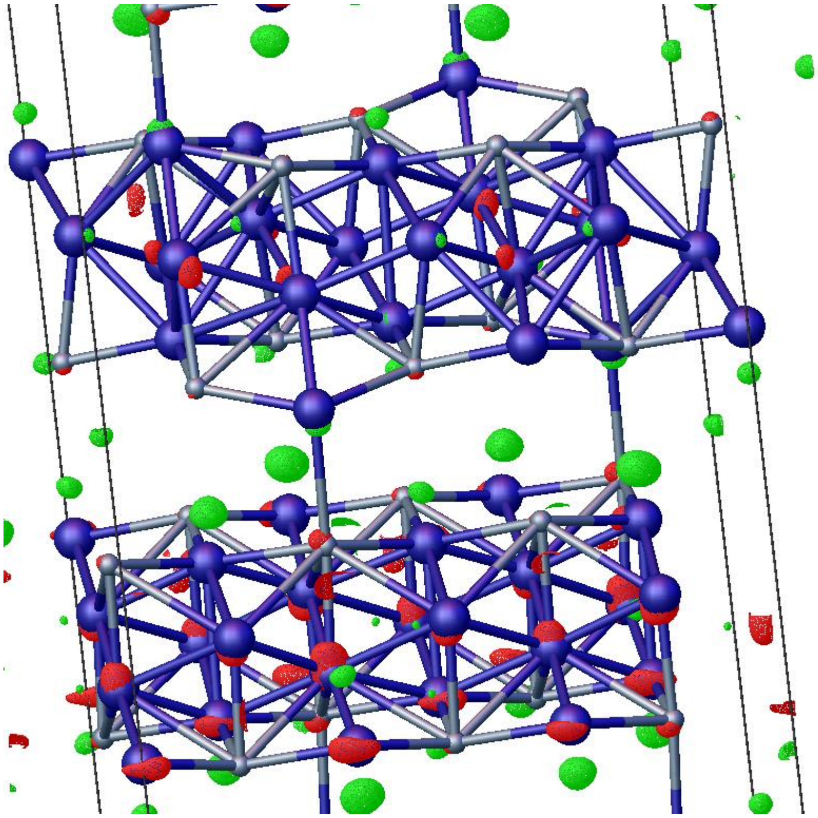

Figure S11 Electron difference map, with a contour of 20 eÅ^{-3}. The green surfaces show areas of electron density missing from the model, and red surfaces indicate the opposite. The structural model used is the ordered model with refined scale, ADP and xyz parameters, fit to all reflections (main + superstructure reflections).

In summary, in order to get a better fit using the present cell and space group C2/c disorder needs to be introduced into the model.

S3.6. Disordered super-structure

Table S7 Coordinates for super-structure based on disordered Cu sites (295 K), a = 7.152(1) Å, b = 12.384(1) Å, c = 28.817(7), β = 108.84(2)º, space group C2/c. Asterisk indicates atoms where the ADPs have been refined anisotropically.

**Atom**	**x/*a***	**y/*b***	**z/*c***	**Uequivalient (Å²)**	Occupancy
Se01	0.2461(4)	0.5026(2)	0.30567(12)	0.0272(6)*	1
Se02	1.2597(4)	0.6689(2)	0.44476(11)	0.0272(6)*	1
Se03	0.7608(4)	0.8346(2)	0.44305(11)	0.0272(6)*	1
Se04	0.7447(4)	0.6677(3)	0.30532(11)	0.0272(6)*	1
Se05	0.7593(4)	0.5004(2)	0.44423(12)	0.0272(6)*	1
Se06	0.2540(4)	0.8356(3)	0.30732(11)	0.0272(6)*	1
Cu01	0.450(3)	0.6685(12)	0.3325(16)	0.024(6)	0.31(7)
Cu02	1.0747(7)	0.8340(4)	0.4285(2)	0.048(2)*	0.878(18)
Cu03	-0.1072(8)	0.8344(5)	0.2847(3)	0.033(2)	0.71(2)
Cu04	0.378(2)	0.543(1)	0.3934(3)	0.042(4)	0.58(4)
Cu05	1.0991(9)	0.6949(5)	0.3568(2)	0.065(3)*	0.897(18)
Cu06	0.787(2)	0.6565(9)	0.3930(3)	0.047(4)	0.58(3)
Cu07	0.6137(12)	0.4620(6)	0.3568(3)	0.043(2)	0.71(1)
Cu08	0.6185(10)	0.6678(6)	0.4622(4)	0.049(3)*	0.69(2)
Cu09	0.7225(13)	0.8389(6)	0.3555(3)	0.046(2)	0.724(11)
Cu10	0.4393(9)	1.0036(4)	0.3234(5)	0.049(4)*	0.79(3)
Cu11	0.4061(11)	0.8030(15)	0.3954(3)	0.047(5)*	0.62(3)
Cu12	1.0744(13)	0.5041(6)	0.4301(6)	0.064(5)*	0.71(3)
Cu13	0.576(3)	0.6724(18)	0.4286(12)	0.043(9)	0.22(3)
Cu14	0.394(5)	1.012(3)	0.2892(18)	0.046(12)	0.16(3)
Cu15	1.127(3)	0.5001(14)	0.4649(11)	0.030(7)	0.23(3)
Cu16	0.305(4)	0.5142(19)	0.3931(6)	0.050(7)	0.33(4)
Cu17	0.404(3)	0.866(3)	0.3954(9)	0.050(9)	0.25(3)
Cu18	-0.083(3)	0.8410(18)	0.3112(13)	0.035(9)	0.20(2)
Cu19	0.868(5)	0.6295(19)	0.3925(7)	0.056(8)	0.31(3)
Cu20	0.429(2)	0.6677(8)	0.3168(13)	0.025(4)	0.48(6)
Cu21	0.616(9)	0.873(6)	0.355(2)	0.046(11)	0.087(15)
Cu22	0.636(11)	0.821(6)	0.347(2)	0.046(11)	0.087(17)
Cu23	0.381(6)	0.686(3)	0.287(2)	0.040(16)	0.12(2)
Cu24	0.672(7)	0.486(4)	0.3503(16)	0.04(1)	0.125(14)
Cu25	0.602(11)	0.554(7)	0.356(3)	0.04(1)	0.06(1)

Table S8 The disordered model used to fit the SCXRD data collected in this work, at room temperature (295 K). *a* = 7.152(1) Å, *b* = 12.384(1) Å, *c* = 28.817(7), *β* = 108.84(2)°, space group C2/c.

Refined parameters	xyz + ADP Isotropic	xyz + ADP anisotropic
**All reflections**		
$N_{Uniq,obs}$	2462	2462
$N_{Parameters}$	113	176
GOF	1.447	1.054
R_1, $R_1[F^2>2\sigma(F^2)]$	0.245, 0.168	0.202, 0.119
wR_2, $wR_2[F^2>2\sigma(F^2)]$	0.514, 0.460	0.355, 0.299
$\Delta\rho_{max}$, $\Delta\rho_{min}$ / e Å^{-3}	8.28, -3.93	2.31, -2.09
**Main reflections only**		
$N_{Uniq,obs}$	236	236
$N_{Parameters}$	113	176
GOF	2.078	1.154
R_1, $R_1[F^2>2\sigma(F^2)]$	0.126, 0.124	0.064, 0.062
wR_2	0.338	0.171
$\Delta\rho_{max}$, $\Delta\rho_{min}$ / e Å^{-3}	6.17, -3.51	1.40, -1.86
**Super-structure reflections only**		
$N_{Uniq,obs}$	2226	2226
$N_{Parameters}$	113	176
GOF	1.652	0.813
R_1, $R_1[F^2>2\sigma(F^2)]$	0.408, 0.285	0.383, 0.253
wR_2	0.607	0.689
$\Delta\rho_{max}$, $\Delta\rho_{min}$ / e Å^{-3}	4.28, -3.43	3.20, -2.74

Table S7 list the atomic positions, occupancies and ADPs for the structural model after modelling disordered Cu sites. Refinement parameters are listed in Table S8, with one model using isotropic ADPs and one using mixed isotropic and anisotropic ADPs.

By introducing disorder into the structure the model nicely fits the main reflections (**R1** = 0.062), while the model do still not fit the super-structure peaks well (**R1** = 0.253), resulting in a total **R1** of 0.119 when using all reflections. It should be noted that the refinements are quite stable with a nice observable to parameters ratio. The misfit is therefore not the result of unstable refinments or too many parameters.

Furthermore, the max/min residual electron density only amount to 2 electrons, indicating that there is no large electron density regions not accounted for (Figure S11). However, the model does not fit the super-structure peak intensities well.

In order to progress and getting a better structural model we believe it is important to use longer exposures focusing on getting better I/σ ratios for all super-structure reflections. In practice this is only feasible using high brilliance synchrotron facilities. From the integration above, 1348 out of the 2226 unique reflections have I/σ < 2.

Better intensities for the super-structure reflections will hopefully allow for a determination of the correct space group symmetry of the super-structure. In the end it might be necessary to use superspace formalism in order to describe the complex super-structure.

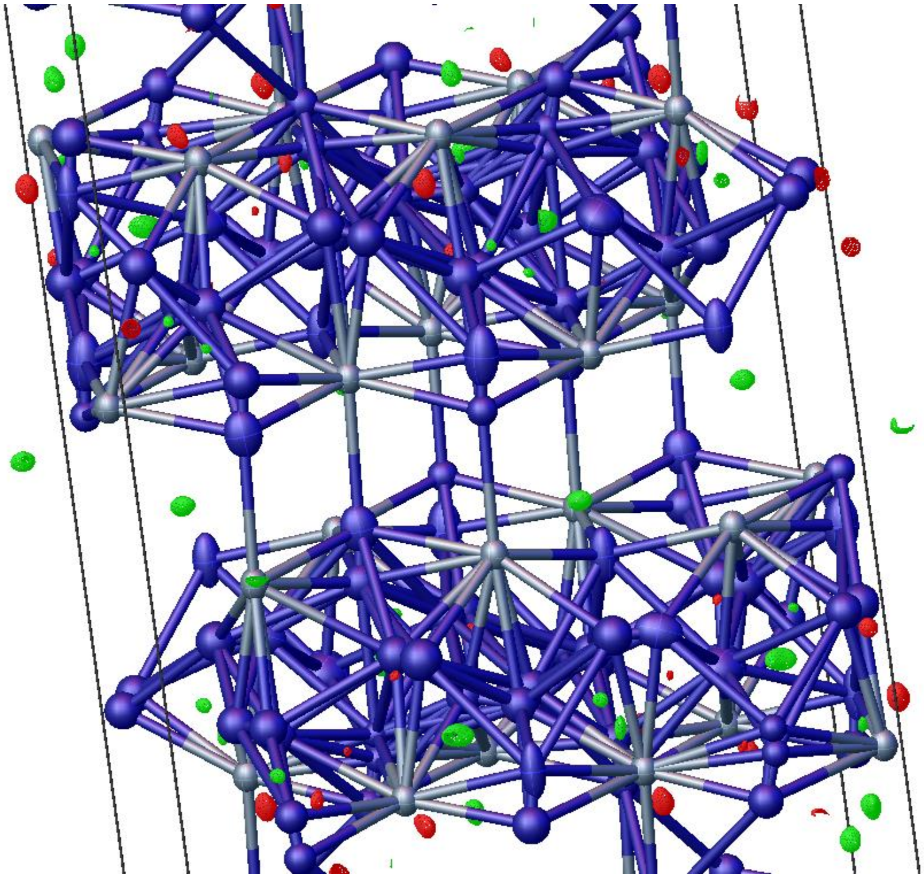

Figure S12 Electron difference map, with a contour of 5 e$Å^{-3}$. The green surfaces show areas of electron density missing from the model, and red surfaces indicate the opposite. The structural model used is a disordered model with refined scale, ADP and xyz parameters, fit to all reflections (main + superstructure reflections).

S4. Supercell reflection intensities, with temperature.

In order to evaluate the intensity of the superstructure peaks we have chosen integration **4**, for the 100 K, 295 K and 372 K data. The large 1x3x4 monoclinic supercell integrates a large number of weak superstructure peaks when the conditions $k/3 \neq n$ and $l/4 \neq n$ are fulfilled.

Table S9 Superstructure peak intensity statistics.

Temp (K)	#reflections with I/σ >3	Average I / σ for super cell reflections (with I/σ >3 at 100 K)	Average I for super cell reflections / I_{max}
100	1408	8.7	0.33 %
295	998	5.7	0.16 %
372	440	2.4	0.06 %

At 100 K integration using cell **4** gives 1408 non-equivalent reflections with $I/\sigma > 3$. 1188 of the reflections are superstructure peaks with an average I/σ of 8.7. It should be noted here that only significant reflections are used to calculate the average and 1130 independent intensities are omitted with $I/\sigma < 3$. The mean intensity of the significant superstructure reflections at 100 K correspond to 0.33 % of the highest intensity reflection. Looking at same reflections at 372 K the value drops to 0.06 % with only 440 independent reflections in total having a $I/\sigma > 3$.

In summary, the intensity of the superstructure peaks decrease with respect to the intensity of the main peaks (a factor 5), as we approach the phase tranition. Thus when approaching the phase transition the superstructure approaches the average structure.

S4.1. Cu occupancies with temperature.

Free refinment of occupancies for Cu1a and b sites are shown in Figure S12.

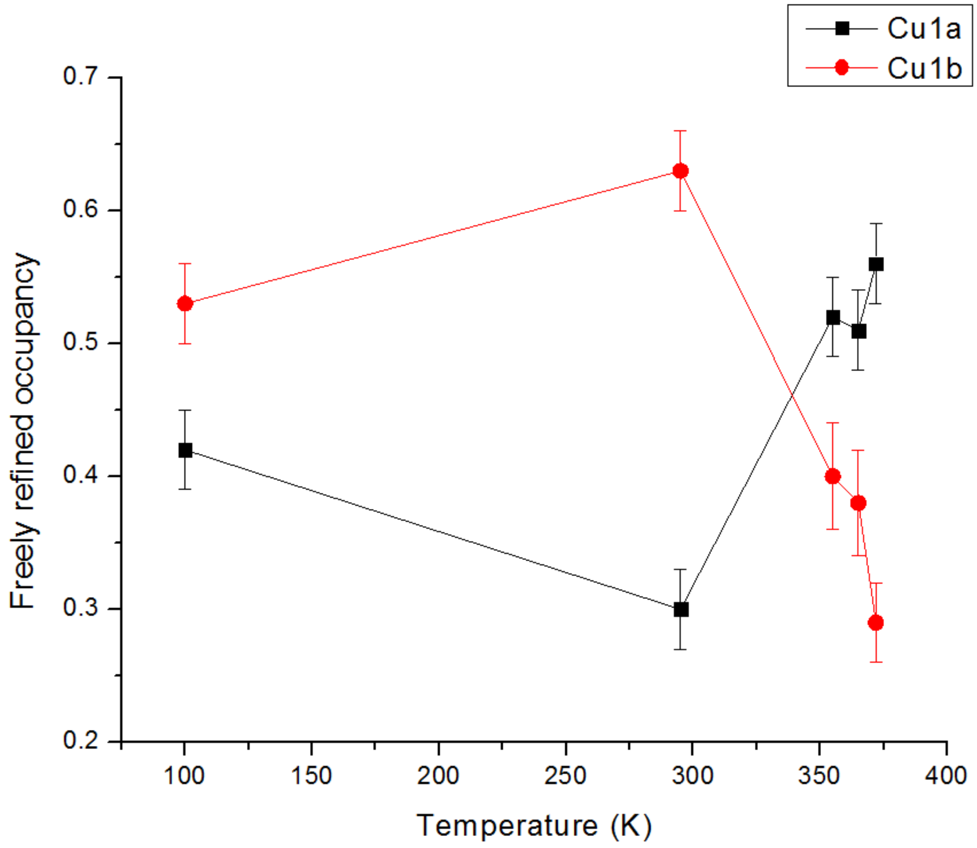

Figure S13 Occupancies for the Cu1a and b site, with temperature. Note that the shown occupancies originate from refinments where the total Cu content is not fixed.

S5. Bärninghausen-tree

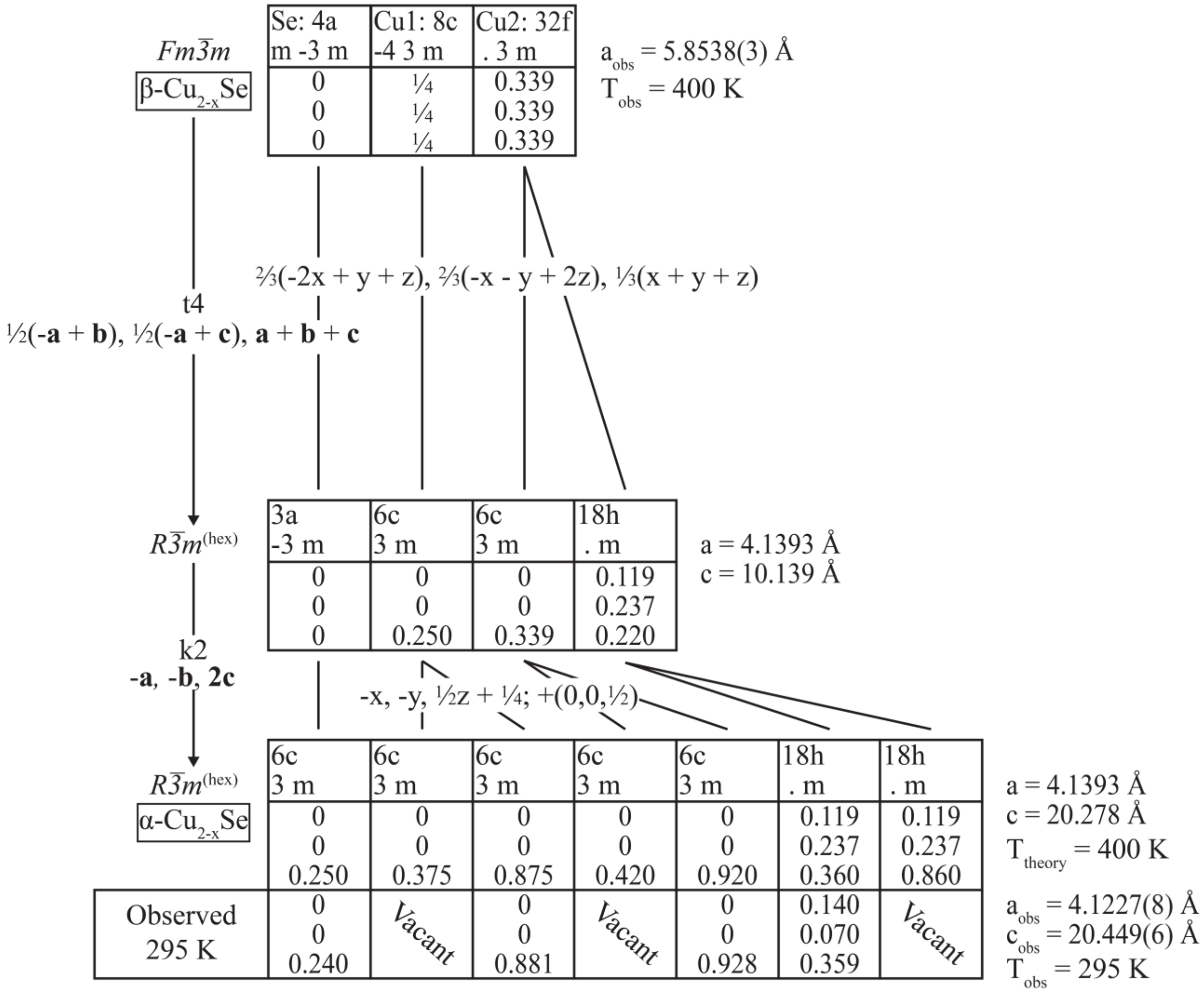

Figure S14Bärninghausen-tree for the two symmetry transformations needed to relate the cubic HT (400K) and the trigonal LT (100 K) structure of $Cu_{2-x}Se$.

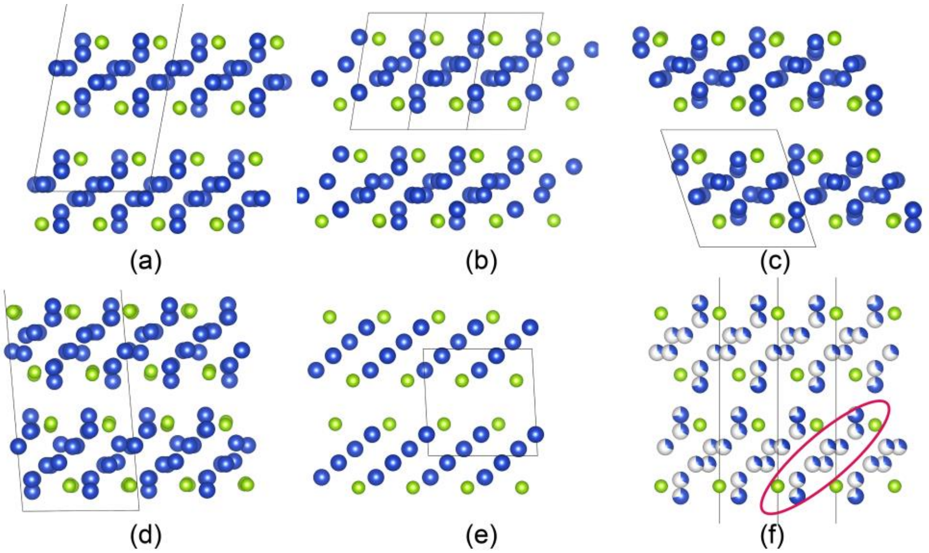

Figure S15 Known structures of β-$Cu_{2-x}Se$; Se and Cu are represented by green and blue spheres, respectively, and the structures have been oriented to show the similarities. a) S1 structure from Lu *et al.*, (2015), with a stabilizing energy of -0.2921 eV compared to the cubic phase. b) S2 structure from Lu *et al.*, (2015), with a stabilizing energy of -0.2920 eV compared to the cubic phase. c) S3 structure from Lu *et al.*, (2015), with a stabilizing energy of -0.2990 eV compared to the cubic phase. d) The structure from Gulay *et al.*, (2011). e) The structure from Nguyen *et al.*, (2013). f) The β-$Cu_{2-x}Se$ structure determined from PXRD Rietveld refinements of data collected at 300 K. The partly colored portion of the Cu atoms indicates the occupancies for the different copper sites. The purple ellipse marks a structural "unit" that can easily be compared and found in all the shown structures.

S6. Synchrotron powder X-ray diffraction

The theoretical diffraction pattern calculated for the structural model obtained by Rietveld refinement is shown in Figure S11 together with the data and difference curves for the 300, 200 and 100 K data.

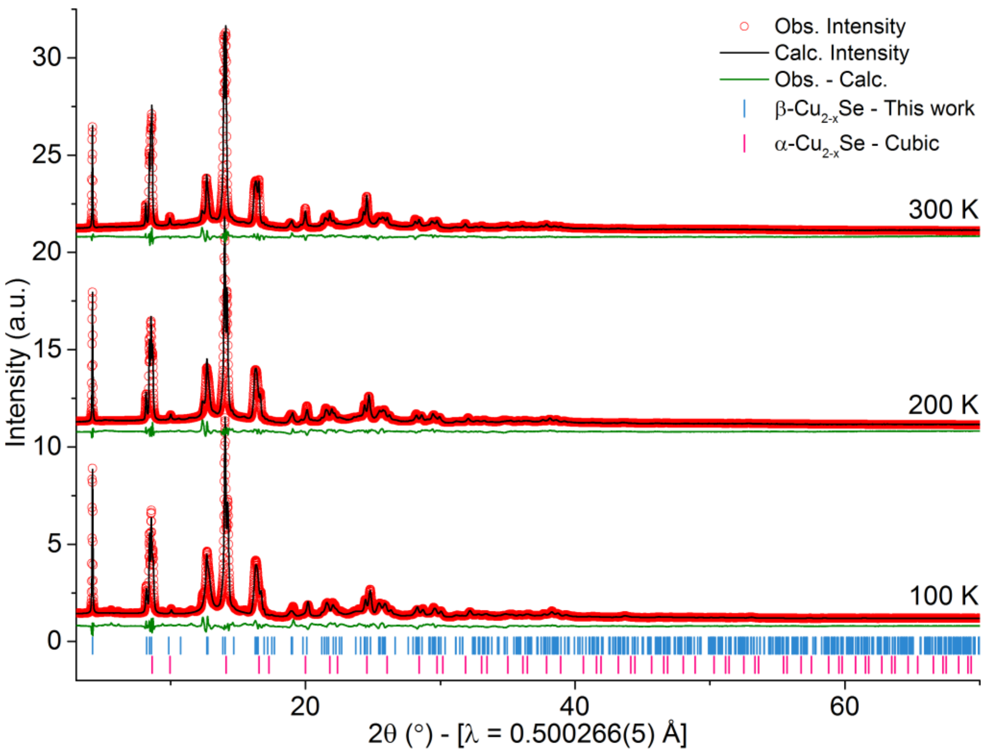

Figure S16 The Rietveld refinements of polycrystalline $Cu_{2-x}Se$ prepared by the bulk method. The cubic phase is from ICSD 150758.

Table S10 Rietveld refinments parameters

	Temp.		(K)	100	200	300
		R_B	(%)	1.875	1.665	1.643
		R_{wp}	(%)	2.961	2.612	2.552
		R_{exp}	(%)	0.820	0.834	0.842
		χ^2		3.610	3.131	3.029
**β-$Cu_{2-x}Se$**	$R\bar{3}m$	a	(Å)	4.0932(2)	4.1035(1)	4.1166(1)
		c	(Å)	20.395(1)	20.404(1)	20.419(1)
		Fraction	(vol %)	79.0(3)	73.5(3)	59.6(3)
	Se	x		2/3	2/3	2/3
		y		1/3	1/3	1/3
		z		0.56999(8)	0.57140(8)	0.57196(9)
		U_{iso}	(Å^2)	0.0076(4)	0.0143(4)	0.0116(4)
	Cu2	x		0.5995(5)	0.5972(5)	0.6047(7)
		z		0.6871(1)	0.6892(1)	0.6870(2)
		U_{iso}	(Å^2)	0.018(1)	0.020(1)	0.032(2)
	Cu1a	x		1/3	1/3	1/3
		y		2/3	2/3	2/3
		z		0.7854(5)	0.7900(5)	0.7875(5)
		U_{iso}	(Å^2)	0.0214(11)	0.0267(11)	0.0269(14)
		Occ.		0.269(4)	0.276(4)	0.328(5)
	Cu1b	z		0.7379(2)	0.7388(2)	0.7390(3)
**α-$Cu_{2-x}Se$**	$Fm\bar{3}m$	a	(Å)	5.7082(2)	5.7296(2)	5.7654(2)
	Se	x = y = z		0	0	0
		U_{iso}	(Å^2)	0.009(1)	0.0183(9)	0.0190(6)

Cu1	x = y = z		1/4	1/4	1/4
	U_{iso}	(Å^2)	0.012(1)	0.025(1)	0.039(2)
	Occ.		0.802(9)	0.807(7)	0.74(3)
Cu2	x = y = z		0.389(8)	0.382(6)	0.318(5)
	Occ.		0.010(2)	0.013(2)	0.040(6)

References

Popa, N. (1998). *J. Appl. Cryst.* **31**, 176-180.

Gulay, L., Daszkiewicz, M., Strok, O. & Pietraszko, A. (2011). *Chem. Met. Alloys* **4**, 200-205.

Lu, P., Liu, H., Yuan, X., Xu, F., Shi, X., Zhao, K., Qiu, W., Zhang, W. & Chen, L. (2015). *J. Mater. Chem. A* **3**, 6901-6908.

Milat, O., Vučić, Z. & Ruščić, B. (1987). *Solid State Ionics* **23**, 37-47.

Nguyen, M.C., Choi, J.-H., Zhao, X., Wang, C.-Z., Zhang, Z. & Ho, K.-M. (2013). *Phys. Rev. Lett.* **111**, 165502.
